# Variation and spread of resistomes in swine manure, manure slurries, and long-term manure-fertilized soils

**DOI:** 10.3389/fmicb.2025.1683394

**Published:** 2025-10-29

**Authors:** Lei Jin, Shujuan Chen, Runmin Kang, Chun Li, Shengzhi Yang, Qiaohui Yang, Ke Zhao, Likou Zou

**Affiliations:** ^1^College of Resources, Sichuan Agricultural University, Chengdu, Sichuan, China; ^2^College of Food Science, Sichuan Agricultural University, Ya’an, Sichuan, China; ^3^Sichuan Animal Science Academy, Chengdu, Sichuan, China; ^4^Sichuan Province Center for Animal Disease Prevention and Control, Chengdu, Sichuan, China; ^5^College of Life Science, Sichuan Agricultural University, Ya’an, Sichuan, China; ^6^Ya’an Quality Inspection of Agricultural Products Monitoring Center, Ya’an, Sichuan, China

**Keywords:** swine manure, anaerobic digestion, ARGs, health risks, horizontal gene transfer

## Abstract

**Background:**

Application of swine manure to soils exacerbates environmental antimicrobial resistance (AMR). However, a comprehensive evaluation of anaerobic digestion’s (AD) mitigation potential against AMR and its influencing factors in swine manure-to-soil systems remains lacking.

**Methods:**

We employed mass spectrometry, metagenomics, and whole-genome sequencing (WGS) to investigate the fate of antibiotics, metals, and antibiotic resistance genes (ARGs) across manures, slurries, and soils from eight pig farms.

**Results:**

Anaerobic digestion reduced antibiotic and metal (except ciprofloxacin) content and risks in manure, but had limited effects on total ARG abundance, while increasing ARG network modularity. High-risk ARG abundance significantly increased from 404.7 in manure to 843.2 in slurries, with health-risk scores rising 1.88-fold during anaerobic digestion. Metagenomic analysis showed metal resistance gene (MRG) diversity and abundance decreased during anaerobic digestion, along with reduced ARG-MRG co-occurrence frequency, whereas mobile genetic element (MGE) diversity and ARG-MGE co-occurrence frequency increased. Escherichia coli was identified as the dominant ARG host. WGS of *E. coli* strains confirmed horizontal gene transfer (HGT) of nine ARGs (e.g., *sul3* and *bla_TEM-1_*), and metagenomics suggested HGT of four ARGs (e.g., *tet*(M)) across different pathogens. Chromium concentrations, bacterial communities and MGEs were significantly associated with ARG profiles. Long-term slurry application resulted in elevated antibiotic, metal, and ARG concentrations in soils, with concomitant increases in high-risk ARGs and health risks.

**Conclusion:**

This study demonstrates AD’s limited effect on mitigating overall ARG abundance and highlights MGEs as critical drivers of ARG maintenance and dissemination from manure to soil process, guiding manure treatment optimization to reduce agricultural AMR risks.

## 1 Introduction

Antimicrobial resistance (AMR) threatens global public health (Sugden et al., 2016). The evolution and dissemination of AMR are largely driven by antibiotic use, primarily through selection pressures that induce bacteria to acquire or develop antibiotic resistance genes (ARGs) (Zhang M. et al., 2021). The widespread use of antibiotics in food-production animals has exacerbated the burden of AMR on human and environmental health, particularly in the pig industry that represents the largest agricultural sector in terms of antibiotic consumption in China (Yan et al., 2024). Various unmetabolized antibiotics are released in swine manure alongside ARGs (Hall et al., 2020). When swine manure is applied as a soil fertilizer, a range of pollutants (e.g., metals and pathogenic microorganisms), particularly manure-borne antibiotics and ARGs are introduced into soil ecosystems, thereby promoting AMR generation and dissemination in soils (Zeng et al., 2024). Thus, mitigation of antibiotics and ARGs in swine manure is necessary prior to land application.

Anaerobic digestion is widely used to remove pollutants from swine manure in intensive farming operations (Pu et al., 2018). Although anaerobic digestion can reduce levels of antibiotics and ARGs in swine manure, significant concentrations often persist in resulting slurries (Zeng et al., 2024). Long-term application of these slurries to agricultural soils can then introduce these contaminants to environments, potentially altering soil microbial communities and increasing antibiotic resistance risks (Liu et al., 2020). Most studies have separately investigated anaerobic digestion or manure/slurry application effects on resistomes, but an integrated evaluation of anaerobic digestion’s mitigation potential for AMR and influence factors in swine manure agricultural systems remains insufficient (Pu et al., 2018).

Despite restrictions on antibiotic use in pig farming, the prevalence of AMR continues to increase in swine manure and related environments (Li X. et al., 2022). This persistence indicates that AMR is not solely driven by antibiotic pressure but involves a complex interplay of various co-selective agents, including heavy metals, disinfectants, and biocides (Pal et al., 2015). Heavy metals, widely utilized as feed additives in livestock production, along with metal pollution from the surrounding environment (e.g., water and air) of pig farms, contribute to elevated levels of heavy metals in swine manure (Peng et al., 2022). These metals impose substantial metal stress that significantly exacerbates the emergence and spread of ARGs through co-selection with metal resistance genes (MRGs) (Wan et al., 2020; Li N. et al., 2022). Furthermore, metals exhibit greater environmental persistence compared to antibiotics due to their non-degradable nature, resulting in prolonged selection pressure within ecosystems such as agricultural soils (Stepanauskas et al., 2005). Consequently, heavy metals are increasingly recognized as major drivers of AMR maintenance and dissemination in agricultural settings (Anedda et al., 2023). However, variations in metal concentrations and their associated environmental risks following the application of swine manure to soils remain insufficiently investigated.

Horizontal gene transfer (HGT) of ARGs and their co-transfer with MRGs via mobile genetic elements (MGEs) lead to increases in the presence of multidrug-resistant bacteria and facilitate AMR (Li et al., 2017). Previous studies related to swine manure investigated correlations among ARGs, MRGs, and MGEs primarily through correlational analyses, which lack location information of genetic elements and cannot accurately judge the co-occurrence of these genetic elements (Zhang H. et al., 2020). Recent metagenomic assembly analyses have revealed co-occurrence patterns among ARGs, MRGs, and MGEs, highlighting the potential mobility of ARGs and their co-selection with MRGs in pig manure and livestock manure-amended greenhouse soils (Li X. et al., 2022; Moura De Sousa et al., 2023). Nevertheless, comprehensive investigations into the co-occurrence of ARGs with MRGs and MGEs in swine manure affected by anaerobic digestion, and the impact of its product slurry application on the co-occurrence of resistance genes in soil, remain scarce. Furthermore, few studies have evaluated HGT events that involve the transfer of potentially mobile ARGs and their co-transfer with MRGs from swine manures to soils.

Comprehensive assessment of the ARG-health risks in swine manure, slurries, and fertilized soils is essential for evaluating anaerobic digestion effectively mitigates AMR risks from swine manure. ARG-ranker framework divided ARGs into four risk categories according to three criteria including enrichment in human-associated environments, gene mobility, and host pathogenicity (Zhang A.-N. et al., 2021). Zhang *et al.,* further estimated an overall health risk of ARGs to humans based on human accessibility, mobility, pathogenicity, and clinical availability (Zhang et al., 2022). These computational frameworks have been successfully applied to assess ARG-related health risks in various environments including the human gut, drinking water, plants, and sludge (Liu et al., 2024). These quantitative frameworks have yet to be applied to assess ARG-related health risks across the swine manure, anaerobic digestion slurry, soil application process.

Sichuan Province, a foremost pig-producing region in China and a national leader in swine inventory, provides an ideal setting to investigate the transfer of antibiotics, metals, ARGs, MRGs, MGEs and pathogen from swine manure to soils, and assess the effectiveness of anaerobic digestion in reducing AMR risks from manure application (Li et al., 2023). In this study, manure, slurry and soil samples were collected from eight large-scale pig farms in the Sichuan Province to (1) quantify and compare the variations in antibiotic and metal concentrations, along with their associated risks, among swine manure, slurry, and between slurry-fertilized and unfertilized soils; (2) characterize the profiles, co-occurrence patterns, and pathogenic hosts of ARGs, MRGs, and MGEs across different stages: during the anaerobic digestion process and in both slurry-fertilized and unfertilized soils, and (3) assess the ARG-related health risks in swine manure, slurry, fertilized soil, and unfertilized soil. These objectives were evaluated using combined liquid chromatography–tandem mass spectrometry (LC/MS/MS), inductively coupled plasma mass spectrometry (ICP-MS), metagenomic, traditional cultivation, and whole-genome sequencing analyses.

## 2 Materials and methods

### 2.1 Study area and sample collection

**Figure 1:**
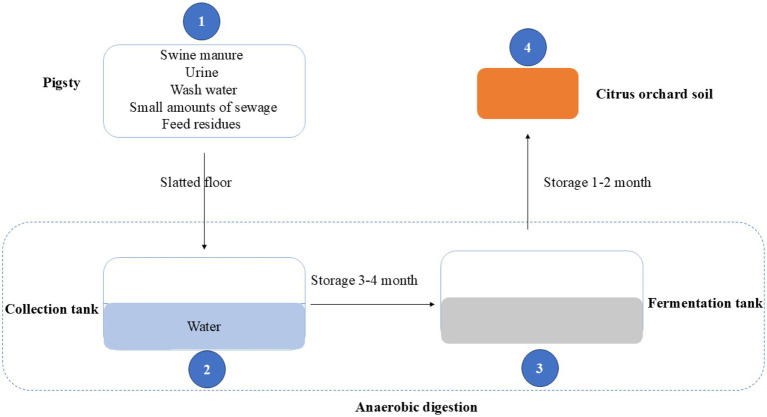
Illustrates the schematic process of anaerobic digestion and specifies the locations where samples were collected for this study. The sampling sites include: (1) fresh pig manure, (2) initial manure slurry (initial anaerobic digestate), (3) slurry (anaerobic digestate), and (4) fertilized soil (amended with the slurry).

This study investigated eight large-scale pig farms in Sichuan Province, China (E 97.35–108.52, N 26.05–34.32), each in operation for over 20 years. These farms annually house between 1,000 and 5,000 pigs, generating 1,100 to 5,500 tons of manure. The land application of swine manure followed the standards DB 14/T 2026—2020 (Shanxi Provincial Administration for Market Regulation, 2020). In brief (Figure 1), swine manure, along with urine, wash water, small amounts of sewage, and feed residues, was collected in a tank pre-filled with 8–10 cm of water for anaerobic digestion. After 3–4 months, the initial manure slurry and residues were discharged from the collection tank and transferred to the fermentation tank for further anaerobic digestion. The resulting slurry was discharged after 1–2 months of storage and had been periodically applied as the sole fertilizer to a citrus orchard soil over a 10-year period. Forest soil samples from Wolong Nature Reserve in Sichuan Province (E 102.87–103.40, N 31.42–34.75) served as the control for fertilized soil.

Fresh swine manure, anaerobically digested slurry, and soil samples were systematically collected in May 2023 following the standardized protocol established by Yan et al. (2024). Fresh manure was obtained from health finishing pigs housed in eight large-scale commercial pig farms in China, where antibiotic use adhered to conventional husbandry practices. All animals were fed a standardized diet primarily composed of corn and soybean. Critically, no antimicrobial agents were administered to any animals within 1 month prior to sampling to eliminate potential confounding effects of recent drug exposure. The corresponding anaerobically digested slurry was collected from the fermentation tank at each respective farm. Slurry-fertilized soil samples (0–20 cm depth) were obtained from citrus orchards that had received slurry amendments for different durations: 10 years (*n* = 2 farms), 15 years (*n* = 2), 20 years (*n* = 2), and 25 years (*n* = 2). To provide a background control, forest soil (0–20 cm depth) was collected from eight distinct locations within the Wolong Nature Reserve in Sichuan Province.

For each individual sample (manure, slurry, fertilized-soil, or forest-soil at each location), material was collected from five sampling sites, thoroughly mixed to obtain a representative composite sample. This yielded a total of 8 composite manure samples, 8 composite slurry samples, 8 composite fertilized-soil samples, and 8 composite forest-soil samples. Notably, slurry refers to the anaerobically digested effluent from fresh swine manure. Each fresh composite sample was divided into three aliquots for specific analyses: approximately 500 g was air-dried for metal determination, another 500 g was stored at 4 °C for *Escherichia coli* cultivation, and the remaining 500 g was immediately frozen at −20 °C for antibiotic residue analysis and DNA extraction.

### 2.2 Determination and risk assessment of antibiotic and metal concentrations

Four major antibiotic classes were quantified: tetracyclines (e.g., tetracycline), fluoroquinolones (e.g., ciprofloxacin, enrofloxacin), *β*-lactams (e.g., ampicillin), and macrolides (e.g., erythromycin). Antibiotics within 1 g of manure, slurry and soil were extracted as previously described (Laconi et al., 2021). Antibiotic concentrations were quantified using an Agilent 6470B triple quadrupole LC–MS/MS (Agilent Technologies Inc., CA, United States). Instrument conditions, method detection limits, limits of quantification, and statistical analyses are detailed in Supplementary Text S1. Arsenic, cadmium, chromium, copper, lead, nickel, and zinc were extracted as previously described (Zhao et al., 2019) and quantified using ICP-MS (Thermo Fisher Scientific Inc., Waltham, MA, United States). The risk quotient (RQ) of antibiotics and the risk index (RI) of metals were calculated following previously described methods (Wang et al., 2024b), as detailed provided (Supplementary Text S2).

### 2.3 Metagenomic sequencing

Total DNA was extracted from samples using DNeasy^®^ PowerSoil^®^ kits (QIAGEN, Germany). Metagenomic sequencing was performed with extracted DNAs at Shanghai Biozeron Biological Technology Co. Ltd. (Shanghai, China) using the Illumina HiSeq X Ten sequencing platform, generating approximately 18 Gb of data per sample. Raw reads were quality-controlled with Trimmomatic v0.39 to remove low-quality bases and adapter sequences (Bolger et al., 2014). Pig (*Sus scrofa*) genomic DNA contamination was removed from the metagenomic data by aligning reads to the *Sus scrofa* reference genome (Sscrofa11.1) using Burrows-Wheeler Aligner (BWA, v0.7.19) (Li and Durbin, 2009). Trimmed metagenomic reads were assembled into contigs using MEGAHIT v1.2.9, followed by open reading frame (ORF) prediction using METAProdigal v2.6.3 (Hyatt et al., 2010; Li et al., 2015). ORFs were clustered into a nonredundant (NR) gene catalog using CD-HIT v4.6.8, and gene abundances were quantified using Salmon v1.10.0, with expression as transcripts per million (TPM) (Fu et al., 2012; Patro et al., 2017). ARGs were annotated against the structured antibiotic resistance genes (SARG) database, while MRGs were annotated against the BacMet database, both using DIAMOND v2.1.8 and BLASTP v2.12.0 alignment strategies (identity ≥ 80%, e-value ≤1 × 10^−5^) (Pal et al., 2014; Buchfink et al., 2015; Yin et al., 2018). MGEs were annotated by aligning sequences to a custom nucleotide MGE database (https://github.com/KatariinaParnanen/MobileGeneticElementDatabase) using BLASTN v2.6.0 (identity ≥ 80%, e-value ≤1 × 10^−5^) (Pärnänen et al., 2018). ARGs and MRGs located on the same contig were defined as co-selected genes, whereas ARGs and MGEs on the same contig were identified as potential mobile ARGs (Li X. et al., 2022). BLASTN v2.6.0 was used to align sequences against the NCBI RefSeq database (≥95% similarity, e-value ≤1 × 10^−9^) to identify hosts of contigs harboring mobile ARGs (Li X. et al., 2022). The homology between contigs was evaluated using BLASTN v2.6.0. High homology was defined as having a minimum similarity of 95%, an alignment covering at least 70% of the shorter contig, a minimum alignment length of 500 bp, and an e-value ≤1 × 10^−5^.

### 2.4 Genomic binning

Genomic binning was performed on the metagenomic assemblies using MetaBAT2 (v2.11.1) to cluster contigs into metagenome-assembled genomes (MAGs) based on sequence composition and coverage depth (Kang et al., 2019). The quality of the resulting MAGs was then assessed with CheckM (Parks et al., 2015). We retained only high-quality MAGs that met the criteria of >70% completeness and <10% contamination. The taxonomic classification of high-quality MAGs was identified using the GTDB-Tk v2.2.6 tool, and pathogen identification was identified using the PHI-base database (Chaumeil et al., 2019). ORFs on each MAGs were predicted using METAProdigal v2.6.3, and the annotation of ARGs, MRGs, and MGEs was performed as described in Section 2.3. The 5 kbp upstream and downstream regions of ARGs where MGEs were present were considered potential mobile ARGs (Zhang et al., 2022).

### 2.5 Isolation and antimicrobial susceptibility testing of *Escherichia coli*

The isolation and measurement of minimum inhibitory concentrations (MICs) of *E. coli* strains were performed following the method described by Tan et al. (2024), described in detail in Supplementary Text S3. Briefly, 25 g of each sample was homogenized with 225 mL of sterile buffered peptone water and pre-enriched at 37 °C for 6 h with shaking at 180 rpm. Subsequently, 1 mL of the pre-enrichment culture was transferred into 9 mL of Enterobacteriaceae Enrichment (EE) broth and incubated at 37 °C for 24 ± 2 h. A loopful of EE broth was streaked onto eosin methylene blue (EMB) agar and incubated at 37 °C for 24 ± 2 h. Presumptive *E. coli* colonies were subcultured on tryptic soy agar (TSA) and incubated overnight at 37 °C. Three colonies per sample were preserved in tryptic soy broth (TSB) containing 20% glycerol at −80 °C. *E. coli* identity was confirmed by 16S rRNA gene sequencing.

Antimicrobial susceptibility testing was conducted using the agar dilution method following Clinical and Laboratory Standards Institute (CLSI) guidelines. The MICs for 12 antibiotics (tetracycline, amoxicillin, ceftriaxone, aztreonam, gentamicin, ciprofloxacin, sulfadiazine, tigecycline, colistin, fosfomycin, erythromycin, and imipenem) were determined. Resistance phenotypes are summarized in Supplementary Table S5.

A total of 39 resistant *E. coli* strains were selected for whole-genome sequencing (WGS) based on sample origin and resistance profiles (Supplementary Table S5). For samples yielding multiple isolates, priority was given to those exhibiting distinct resistance phenotypes. When varied resistance patterns were observed within a sample, isolates with the broadest resistance spectrum were chosen, with a maximum of three isolates selected per sample.

### 2.6 Second-generation whole genome sequencing

Genomic DNA from *E. coli* strains was extracted using the Bacterial Genome Kit (TIANGEN, Beijing, China) and WGS was performed at Shanghai Biozeron Biological Technology Co., Ltd. (Shanghai, China) on the Illumina HiSeq 2,500 platform. Low-quality reads were removed using Trimmomatic v0.39, and *de novo* genome assembly was conducted with SPAdes v4.0.0 (Bankevich et al., 2012; Bolger et al., 2014). The annotation of ARGs, MRGs, and MGEs was conducted as described in Section 2.3. ARGs with MGEs present in either 5 kbp upstream or downstream regions were identified as potential mobile ARGs for further analysis (Zhang A.-N. et al., 2021). Pan-genome analysis of *E. coli* strains isolated from the same pig farm was conducted using the Roary software program (Liu et al., 2022). Multiple sequence alignment was performed using the Multiple Alignment using Fast Fourier Transform (MAFFT) tool and phylogenetic trees were generated using IQ-TREE v2.3.6 (Minh et al., 2020). Phylogenetic trees were visualized using the Interactive Tree of Life (iTOL) platform (Letunic and Bork, 2021).

### 2.7 Health risk assessment of ARGs

ARG risk was evaluated using the Arg_ranker v2.0 software program (Zhang A.-N. et al., 2021). Health risk assessments of ARGs were conducted as previously described (Zhang et al., 2022), with detailed methods provided (Supplementary Text S4).

### 2.8 Statistical analyses

Significant differences in measurements between the manure and slurry groups were tested using paired *t*-tests and Wilcoxon signed-rank tests with the GraphPad Prism v10.1.2 program. Independent *t*-tests and Mann–Whitney U tests were also used to compare measurements between fertilized and control soil groups. Diversity analysis (based on the Shannon diversity index), principal coordinates analysis (PCoA), permutational multivariate analysis of variance (PERMANOVA), and distance-based redundancy analysis (dbRDA) were conducted using the vegan package for R v4.3.0. Correlation coefficients were calculated using the psych package for R v4.3.0. Network visualization and topology analysis were conducted using the Gephi v0.9.2 software program. Modularity was computed using the igraph package for R v4.3.0. Community assembly processes were analyzed using the NST package for R v4.3.0 while employing a null model. Other visual analyses were conducted using the ggplot2 package for R v4.3.0.

## 3 Results

### 3.1 Concentrations and risk assessments of antibiotics

**Figure 2:**
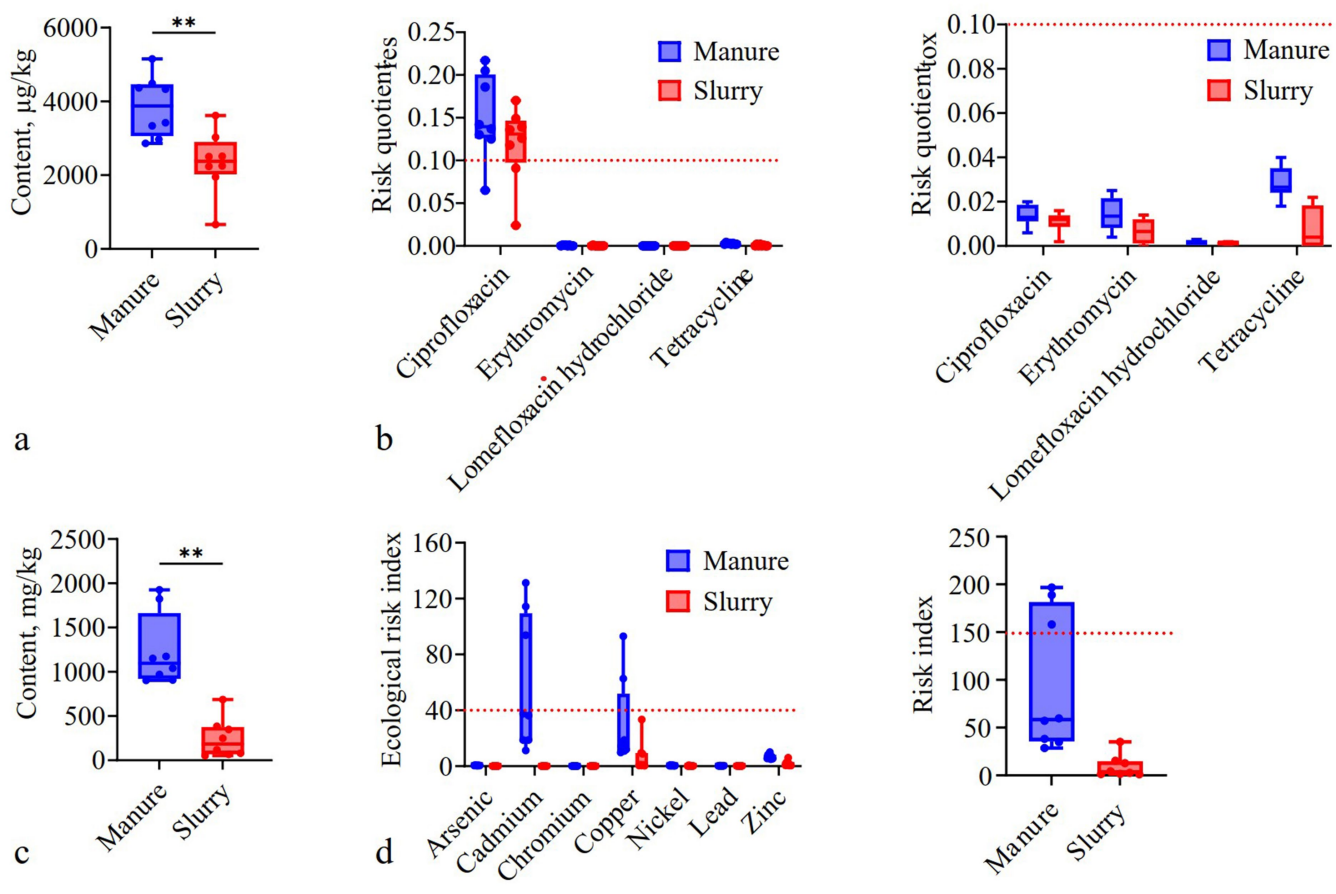
The concentrations and risks of antibiotics and metals in swine manure and anaerobic digestion slurries. **(a)** Total antibiotic concentrations (**: *p* < 0.01); **(b)** Antibiotic resistance and toxicological risk (the red dashed lines indicate the 0.1 threshold). **(c)** Total metal concentrations (**: *p* < 0.01). **(d)** Metal ecological and toxicological risk (the red dashed lines indicate the 40 and 150 thresholds, respectively).

Ciprofloxacin exhibited the highest average concentrations in both the manure (1,746.63 μg/kg) and slurry (1,316.54 μg/kg) samples (Supplementary Table S1). Total antibiotic concentrations were significantly higher in manure (average: 3,457.19 μg/kg) than in slurries (2,037.11 μg/kg) (*p* < 0.05) (Figure 2a). Specifically, concentrations of ampicillin, tetracycline, erythromycin, and lomefloxacin were significantly lower in the slurry samples compared to the manure samples (*p* < 0.05), with removal rates of 100, 60.94, 56.92, and 30.8%, respectively (*p* < 0.05) (Supplementary Figure S1). Over 75% of manure and slurry samples exhibited moderate antibiotic resistance risk for ciprofloxacin (RQres: 0.1–1), while erythromycin, lomefloxacin, and tetracycline exhibited low resistance risks (RQres <0.1) across all samples (Figure 2b). All manure and slurry samples exhibited low toxicological risk (RQtox <0.1) (Figure 2b).

Slurry application increased antibiotic concentrations in soils, with ciprofloxacin, erythromycin, lomefloxacin, and tetracycline detected in fertilized soils, but not control soils (Supplementary Table S1). Ciprofloxacin and tetracycline were the most abundant antibiotics in fertilized soils, with concentrations ranging from 338.71 to 1,999.44 μg/kg and 164.03 to 213.69 μg/kg, respectively. Among them, ciprofloxacin exhibited high antibiotic resistance and toxicological risks in fertilized soils (RQres and RQtox >1) in over 85% of samples (Supplementary Figure S2). Lomefloxacin, erythromycin, and tetracycline showed moderate resistance (RQres: 0.1–1) in 12.5, 25, and 37.5% of samples, respectively. Additionally, 37.5% of samples exhibited high toxicological risk for tetracycline (RQtox > 1), while 87.5% of samples exhibited moderate risk for lomefloxacin (RQtox: 0.1–1).

### 3.2 Concentrations and risk assessments of metals

Zinc and copper exhibited the highest average concentrations in both manure (593.08 and 191.57 mg/kg) and slurry (383.74 and 34.69 mg/kg) samples (Supplementary Table S2). Total metal concentrations were significantly higher in manure (average: 1,236.81 mg/kg) than in slurries (248.66 mg/kg) (Figure 2c). Metal concentrations were significantly lower in slurry samples than in manure samples (*p* < 0.05), with removal rates following the order of: cadmium (100%) > arsenic (95.25%) > copper (85.64%) > zinc (73.33%) > nickel (46.46%) > lead (29.32%) > chromium (21.00%) (Supplementary Figure S3). The permissible heavy metal concentration thresholds set by the swine slurry land application standard (DB 14/T 2026—2020) were not exceeded in any of the slurry samples. Ecological risk index (ERI) values for all metals were generally < 40 in all manure and slurry samples (Figure 2d), except for cadmium and copper in some manure samples. ERI values for all metals were significantly higher in manure than slurry samples (*p* < 0.05), except for chromium. The overall RI values for the seven metals was significantly lower in slurry samples (<150) than in manure samples (*p* < 0.05), with 37.5% of manure samples having RI values between 150 and 300 (Figure 2d).

In soil, zinc concentrations were highest in both fertilized (average: 72.86 mg/kg) and control (62.23 mg/kg) soil samples (Supplementary Table S2). Nickel, cadmium, and lead concentrations were significantly higher in the fertilized soils, while chromium and arsenic concentrations were higher in the control soils (*p* < 0.05) (Supplementary Figure S4). All metal concentrations in soils remained below the background threshold for the Sichuan region (Supplementary Table S3). Both ERI and RI values for all metals remained consistently below 40 and 150, respectively, regardless of slurry addition (Supplementary Figure S5).

### 3.3 ARG variation

A total of 3,511,263,714 raw reads were obtained from the 32 samples through metagenomic sequencing. After quality filtering and adapter removal, 3,424,973,062 clean reads were retained for subsequent analysis. Assembly of these high-quality reads yielded 13,556,366 contigs.

**Figure 3:**
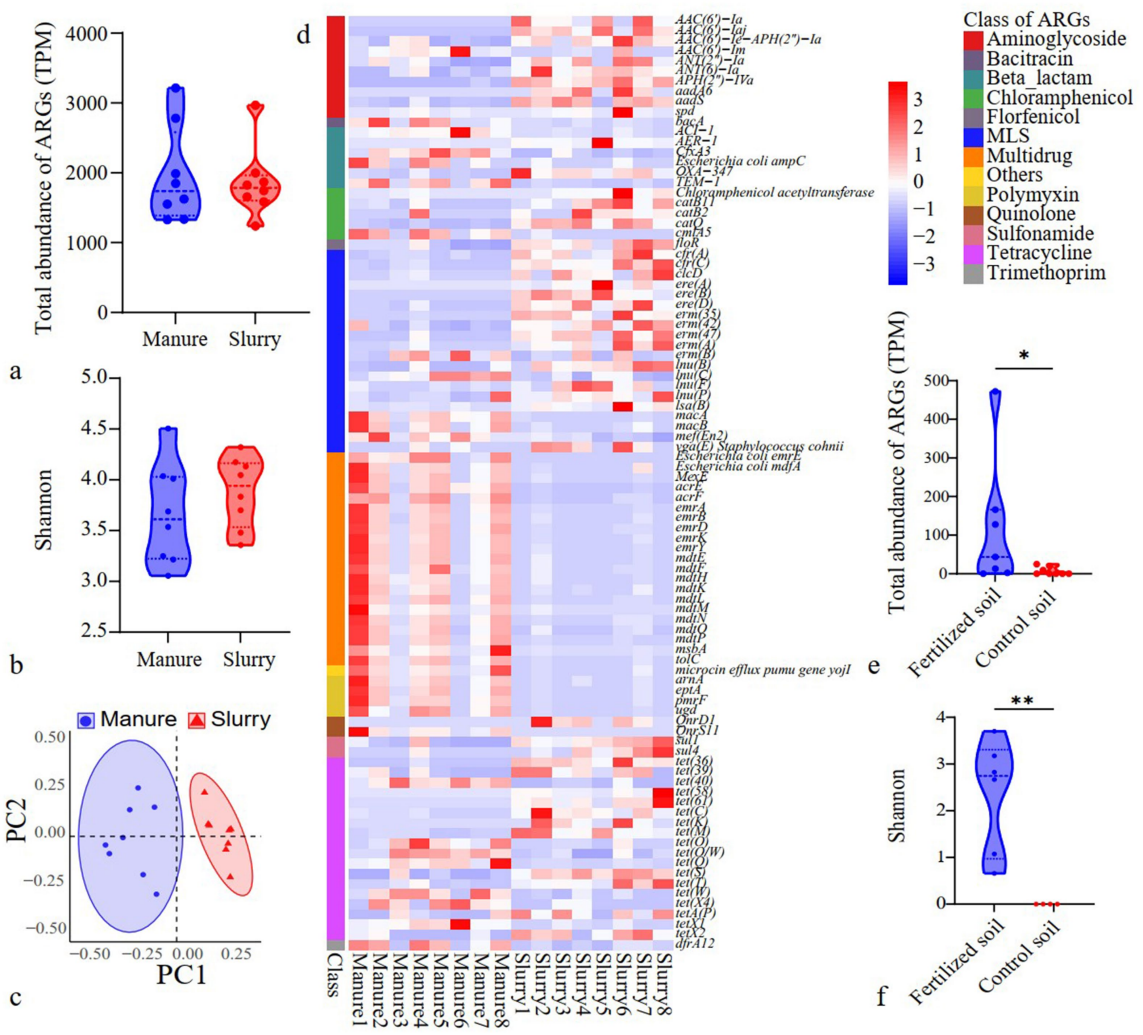
Variation of ARGs in swine manure, anaerobic digestion slurries, and soils. **(a)** Comparison of ARGs identified in manure and slurries; **(b)** Diversity of ARGs in swine manure and slurries; **(c)** PCoA of ARG profiles from manures and slurries; **(d)** Differentially abundant ARGs in manure and slurry samples (*p* < 0.05); **(e)** Total abundances of ARGs in soil (*: *p* < 0.05); **(f)** Diversity of ARGs in soils (**: *p* < 0.01).

Significant differences were not observed in the total abundances and diversity of ARGs when comparing manure and slurry groups (*p* > 0.05) (Figures 3a,b). Tetracycline, macrolide-lincosamide-streptogramin (MLS), and aminoglycoside resistance genes were the predominant ARG types in both manure and slurry samples (Supplementary Figure S6). At the ARG subtype level (Figure 3c), significant compositional differences were observed between manure and slurry sample profiles (*p* < 0.05). A total of 92 ARGs exhibited significantly different abundances between manure and slurry samples (*p* < 0.05) (Figure 3d). Specifically, ARG subtypes associated with multidrug resistance exhibited significantly lower abundances in slurry samples, while those related to tetracycline, aminoglycosides, and MLS resistance exhibited significantly higher abundances (*p* < 0.05). Network analysis revealed higher linkage density and clustering coefficients for ARG networks in manure group (Supplementary Figure S7). However, ARG network modularity was higher for slurry group (0.606) than for manure group (0.567).

Slurry application significantly increased the total abundances and diversity of ARGs in soils (*p* < 0.05) (Figures 3e,f). In fertilized soils, ARGs primarily comprised those related to aminoglycoside, bacitracin, multidrug, sulfonamide, and tetracycline resistance, while rifamycin- and trimethoprim-related ARGs were most prevalent in control soils (Supplementary Figure S8). Notably, 78.0% of ARG subtypes in fertilized soils overlapped with those in slurries, while no ARGs in control soils were observed in slurry samples (Supplementary Figure S9). Network and community assembly analyses of ARGs in soil samples were not conducted due to the limited number of ARGs (*n* = 4) identified in control soils.

### 3.4 Co-occurrence and potential HGT of ARGs with MRGs and MGEs

#### 3.4.1 Analysis based on metagenomic sequencing

**Figure 4:**
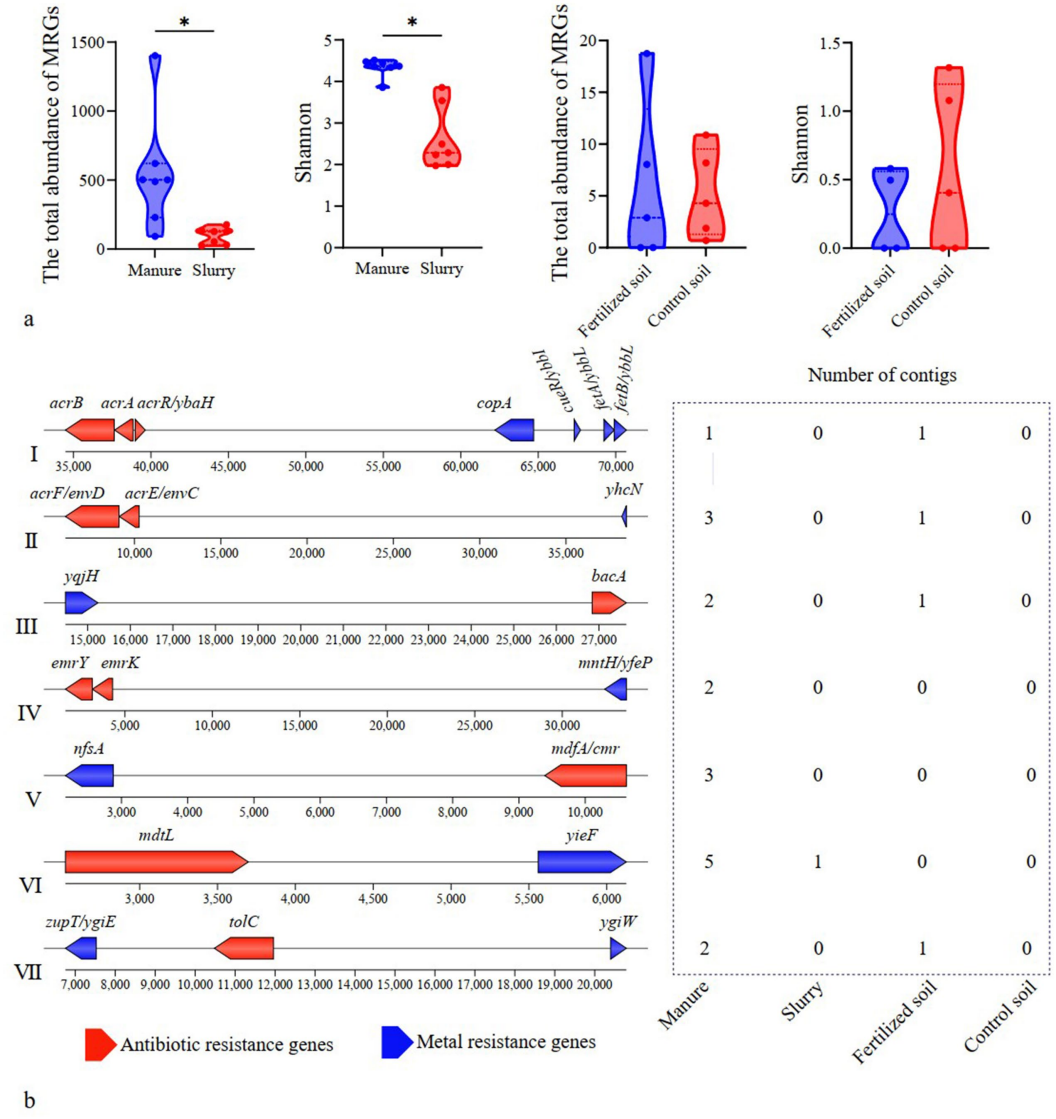
Variations in metal resistance genes (MRGs) and their co-occurrence with antibiotic resistance genes (ARGs). **(a)** Total abundance and diversity of MRGs in swine manure, slurry, and soil samples; **(b)** Arrangement of antibiotic and metal resistance genes that co-occurred in representative contigs. Only those co-occurrences present in at least two different contigs are shown.

MRGs in manure primarily conferred resistance to multi-metals, copper, and zinc, in slurry were predominantly associated with mercury and copper resistance, and those in fertilized and control soils were both primarily resistant to multi-metals (Supplementary Figure S10). The total abundance and diversity of MRGs was significantly higher in manure than in slurry (*p* < 0.05), whereas no significant effect was observed between fertilized and control soils (*p* > 0.05) (Figure 4a).

Twenty-three assembled contigs encoded both ARGs and MRGs, comprising 7 distinct co-occurrence subtypes that involve 11 ARGs and 11 MRGs (Figure 4b). Multidrug and multi-metal resistance genes were the most frequently co-occurring genes. All co-occurrence subtypes were identified in manure samples, while only one subtype (the co-occurrence of *mdtL* and *yieF*) was observed in the slurry samples. Four co-occurrence subtypes were detected in fertilized soils, while no ARG-MRG co-occurrences were detected in control soils (Figure 4b).

**Figure 5:**
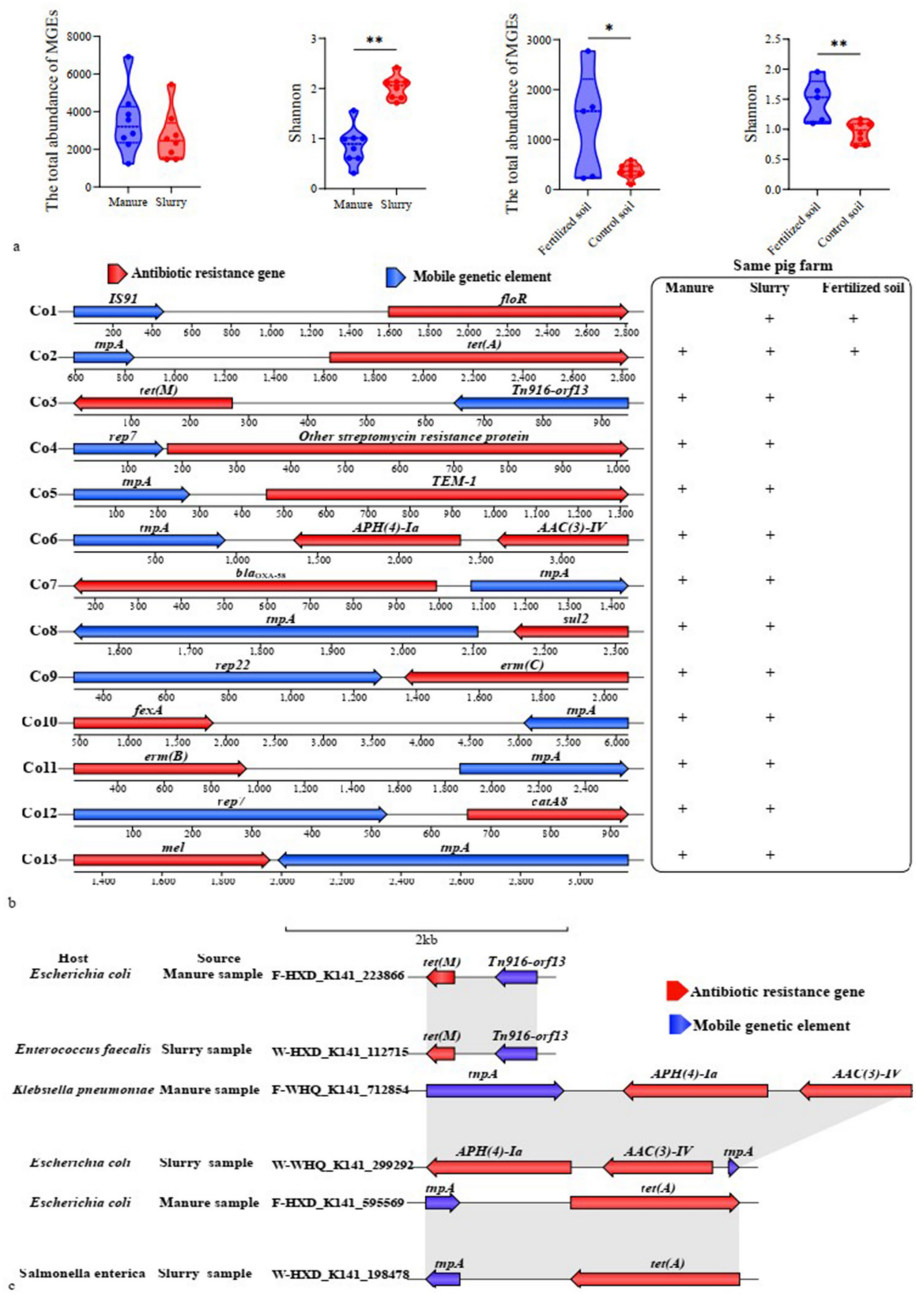
Mobility and HGT potential of ARGs. **(a)** Variation of MGE abundance and diversity; **(b)** Shared ARG-MGE co-occurrence patterns between swine manure-slurry and slurry-soil comparisons within the same pig farm (“+” indicates co-occurrence patterns were detected in the respective sample types); **(c)** Host identification and homology analysis of contigs carrying identical ARG-MGE co-occurrence patterns in manure and slurry samples from a pig farm.

Transposases were the most prevalent MGEs in manure, slurry, and fertilized soil, whereas the insertion sequence IS91 was predominant in control soil (Supplementary Figure S11). Although no significant difference was observed in the total abundance of MGEs between manure and slurry (*p* > 0.05), MGE diversity was significantly higher in slurry than in manure (*p* < 0.05) (Figure 5a). In soil samples, both the total abundance and diversity of MGEs were significantly greater in fertilized soil compared to control soil (*p* < 0.05) (Figure 5a).

A total of 162 contigs were found to encoded both ARGs and MGEs, none of which contained any MRGs (Supplementary Figure S12; Supplementary Table S4). Among these contigs, 71, 83 and 8 were identified in manure, slurry, and fertilized soils, respectively. The co-occurrence events formed 37 distinct patterns involving 34 ARGs that were classified as potential mobile ARGs that primarily conferred resistance to MLS, aminoglycosides, and tetracycline. Of these, 28, 34, and 7 potential mobile ARGs were detected in manure, slurry, and fertilized soil samples, respectively, while no such ARGs were identified in control soils.

Thirteen co-occurrence patterns were observed as shared between manure and slurry groups within the same pig farms, while 2 co-occurrences patterns were common to slurry and fertilized soil groups (Figure 5b). To assess whether the potential mobile ARGs within shared co-occurrence patterns across different sample sources resulted from HGT, comparative sequence homology analysis and host identification were conducted. Specifically, contigs were queried that contained identical co-occurrence events across manure-slurry or slurry-fertilized soil pairs within each farm. HGT events were inferred when contigs from distinct sources exhibited high sequence homology, but were phylogenetically linked to distinct species. Using this approach, *tet*(M), *APH(4)-Ia*, *AAC(3)-IV*, and *tet*(A) likely underwent HGT between different pathogens involved in anaerobic digestion (Figure 5c). Specifically, a homologous sequence carrying *tet*(A) and *tnpA* was identified in *E. coli* from a manure sample and in *Salmonella enterica* from a slurry sample. Similarly, a homologous sequence containing *AAC(3)-IV*, *APH(4)-Ia*, and *tnpA* was detected in *Klebsiella pneumoniae* from a manure sample and in *E. coli* from a slurry sample. Further, a homologous sequence with *tet(M)* and *Tn916-orf13* was identified in *E. coli* from manure and in *Enterococcus faecalis* from slurry. Coincidently, the relative abundance of *tet(M)* in the slurry group was significantly higher than in the manure group. HGT and persistence could not be distinguished for the remaining potential mobile ARGs because high sequence homology was consistently linked to identical host species, and a species-level phylogenetic tree was absent in the metagenomic analysis.

#### 3.4.2 Analysis based on WGS of isolates

**Figure 6:**
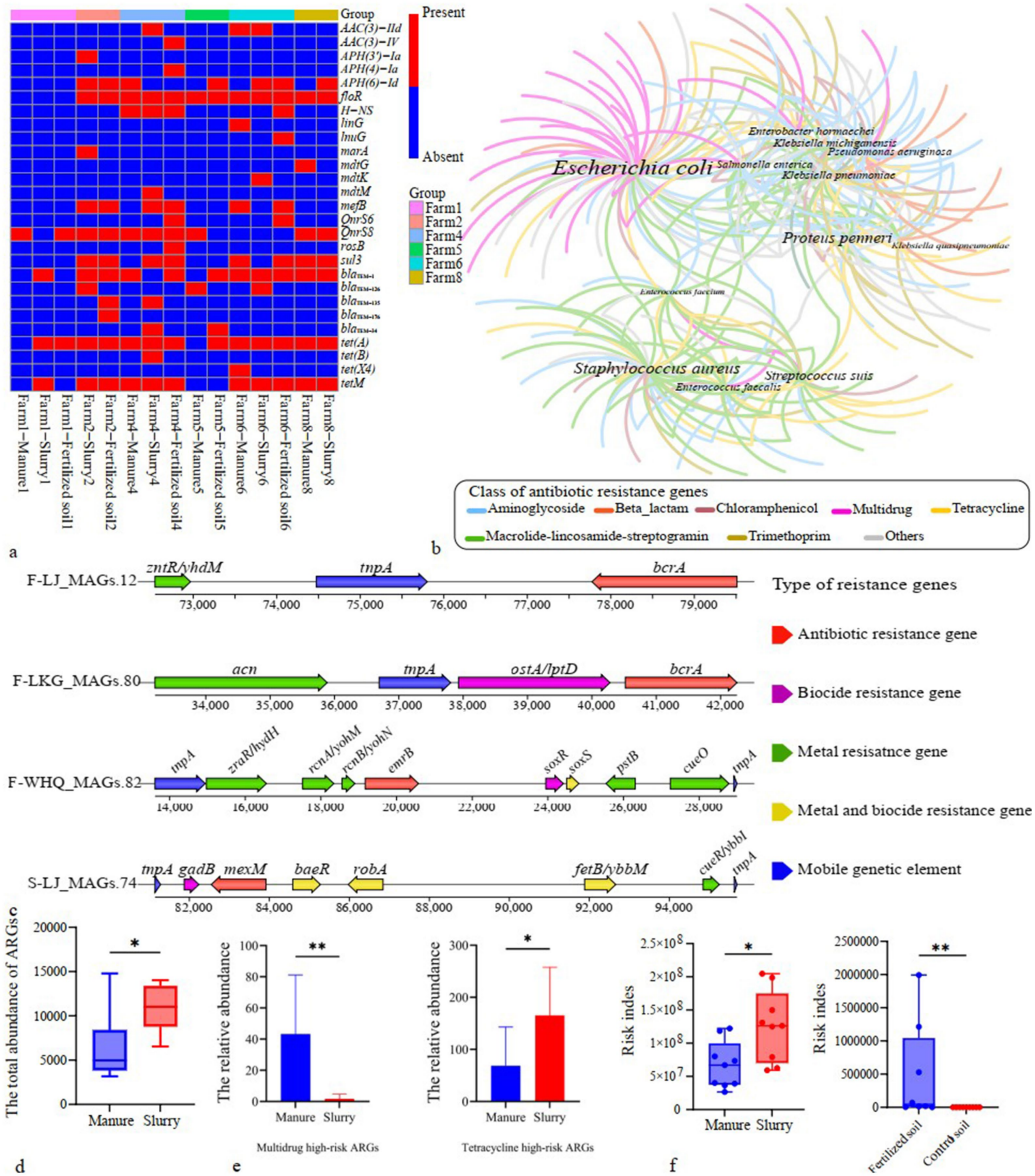
Host association and health risk assessment of antibiotic resistance genes (ARGs). **(a)** Potential mobile antibiotic resistance genes (ARGs) distribution in *E. coli*; **(b)** Co-occurrence network analysis of dominant bacterial hosts carrying ARG-contigs (word size corresponds to the diversity of encoded ARG subtypes); **(c)** Genomic context of potentially mobile ARGs alongside neighboring resistance genes and mobile genetic elements (MGEs) in metagenome-assembled-genomes (MAGs); **(d)** Abundances of high-risk ARGs in swine manure and slurry samples (*: *p* < 0.05); **(e)** Relative abundances of multidrug and tetracycline high-risk antibiotic resistance genes (ARGs) in swine manure and slurry samples; **(f)** Health risk indices of ARGs identified in swine manure, slurry, and soil samples (*: *p* < 0.05; **: *p* < 0.01).

**Figure 7:**
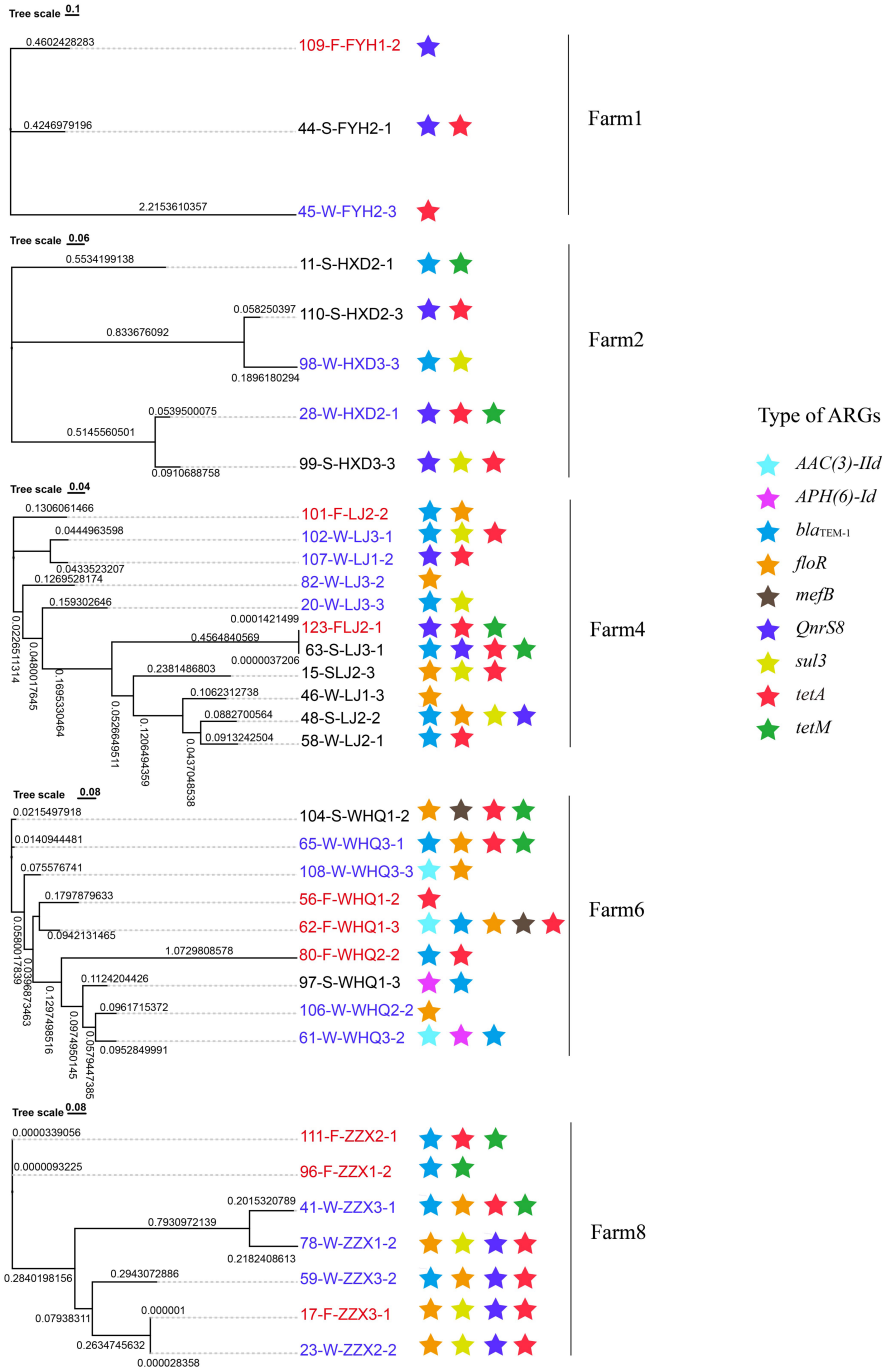
Phylogenetic relationships of *E. coli* strains isolated from swine manure (red labels), slurry (blue labels), and fertilized soils (black labels). Colored stars (★) indicate strains carrying homologous sequences of specific antibiotic resistance genes (ARGs). Strain labels are color-coded by source, with red indicating swine manure, blue indicating slurries, and black indicating fertilized soils.

*E. coli* was the predominant pathogenic host of potential mobile ARGs in manure, slurry, and fertilized soil samples, consequently representing an ideal species to investigate HGT of potential mobile ARGs within the same bacterial species with different sample sources (See section 3.5). A total of 39 resistant *E. coli* strains were obtained from manure (*n* = 10), slurry (*n* = 18), and fertilized soil (*n* = 11) samples that were subjected to WGS, leading to the identification of 27 potential mobile ARGs across all isolates (Figure 6a; Supplementary Table S5). Of these, 8 potential mobile ARGs were shared between manure and slurry samples from the same pig farm, while 9 were shared between slurry and fertilized soil samples from the same farm. To investigate the origins and potential HGT of these shared mobile ARGs, gene tree or BLAST-based sequence alignment analyses were conducted for each ARG from the same pig farm. If a gene was persistent without any HGT, the topology of the gene tree would align with the phylogenetic tree of *E. coli* strains that was constructed using informative genetic variation including core single nucleotide polymorphisms (SNPs) extracted from a core genome alignment. Based on this framework, 7 shared potential mobile ARGs (*AAC(3)-IId*, *sul3*, *bla_TEM-1_*, *tet*(A), *tet*M, *floR*, *QnrS8*) were likely subjected to HGT between *E. coli* species during anerobic digestion, while 8 shared potential mobile ARGs (*APH(6)-Id*, *sul3*, *bla_TEM-1_*, *tet*(A), *tet*M, *floR*, *mefB*, *QnrS8*) were likely subjected to HGT between *E. coli* species in slurry fertilized soils (Figure 7).

### 3.5 Host and health risks of ARGs

Comparison of assembled contig sequences against the NCBI RefSeq database revealed 902 species as potential hosts of ARG-carrying contigs. Among these, 448, 504, 200, and 9 species were identified in manure, slurry, fertilized soil, and control soil samples, respectively. *E. coli*, *Staphylococcus aureus*, *Proteus penneri*, and *Streptococcus suis* were the dominant hosts of ARG-carrying contigs across all groups (Supplementary Figure S13). Multidrug resistance genes were the most prevalent ARGs carried by *E. coli*, followed by those encoding aminoglycoside, MLS, and tetracycline resistance genes. *S. aureus*, *P. penneri*, and *S. suis* predominantly carried aminoglycoside, MLS, and tetracycline resistance genes (Figure 6b). *E. coli* was the most prevalent pathogen in both the manure and slurry groups, while *S. suis* was also highly abundant in the slurry group (Supplementary Figure S14). Analysis of genomes generated from metagenomic datasets identified 389 species as hosts of ARGs, with *E. coli* being the most predominant (Supplementary Figure S15). The relative abundance of *E. coli* was significantly higher in manure samples (*p* < 0.05), whereas *S. suis* was more abundant in slurry samples (*p* < 0.05) (Supplementary Figure S16). In fertilized soils, *E. coli* was the major identified pathogen, but was absent in the control soils (Supplementary Figure S14).

The hosts of 21 potential mobile ARG-carrying contigs were notably identified as pathogenic bacteria. In addition, metagenomic binning identified a pathogenic MAG (*E. coli*) that harbored four potential mobile ARGs (*bcrA*, *emrB*, *mexM*, and *vanH_in_vanP_cl*) (Figure 6c). A total of 52 potential mobile ARGs encoded by pathogenic bacteria were identified from genome-resolved metagenomic analyses and WGS of *E. coli* strains (Supplementary Table S6). Among these, 25 ARGs that were previously classified as non-Rank I based on the ARG Ranker platform should now be reclassified as Rank I ARGs.

ARG ranker analysis combined with Rank I ARGs identified in our study, indicated that 74, 82, and 60 Rank I ARGs were identified in the manure, slurry, and fertilized soil samples, but none were detected in control soils. Aminoglycoside, MLS, tetracycline, and sulfonamide resistance genes were the most prevalent Rank I ARGs identified in the manure and slurry samples, while sulfonamide, aminoglycoside, bacitracin, and florfenicol resistance genes were the most prevalent Rank I ARGs in fertilized soils (Supplementary Figure S17). The total abundance of Rank I ARGs was significantly greater in slurry (843.2) than manure (404.7) samples (*p* < 0.05) (Figure 6d). Specifically, multidrug-resistant Rank I ARGs were significantly more abundant in manure samples, whereas tetracycline-resistant Rank I ARGs were more abundant in slurry samples (*p* < 0.05) (Figure 6e). A total of 16 Rank I ARG subtypes exhibited significant differences in abundances between manure and slurry samples (*p* < 0.05) (Supplementary Figure S18). Of these, 8 ARGs were enriched in manure samples and these were primarily multidrug ARGs. The other 8 ARGs were significantly enriched in slurry samples, including aminoglycoside, MLS, tetracycline (*tet(M)*) and florfenicol (*floR*) resistance ARGs. Approximately 97.0% of Rank I ARGs in fertilized soils overlapped with those in the slurry group.

To comprehensively assess health risks posed by ARGs in manure, slurries, and soils, factors like human accessibility, mobility, pathogenicity, and abundances were integrated to calculate a risk index for the ARGs. The risk index values of ARGs in the slurry samples were significantly higher than in the manure group (*p* < 0.05) (Figure 6f). Furthermore, the risk index values of ARGs in fertilized soils were significantly higher than in control soils (*p* < 0.05) (Figure 6f).

### 3.6 Factors influencing ARG profiles

PERMANOVA analysis identified chromium concentrations (*R*^2^ = 0.237, *p* < 0.05) and bacterial community composition (*R*^2^ = 0.255, *p* < 0.05) as the primary factors influencing ARG profiles in the manure samples. Likewise, chromium concentrations (*R*^2^ = 0.300, *p* < 0.05) and bacterial community composition (*R*^2^ = 0.367, *p* < 0.05) were also highly related to slurry sample ARG profiles, alongside MGEs (*R*^2^ = 0.289, *p* < 0.05). To further quantify the contributions of key factors to ARG profile variation, dbRDA was performed (Supplementary Figure S19). The first two dbRDA axes explained 44.66 and 53.73% of the variation in ARG profiles in the manure and slurry groups, respectively. Chromium was the most significant factor associated with ARG profiles, accounting for 23.73 and 30.00% of variation in ARG profiles in the manure and slurry samples, respectively. In contrast, PERMANOVA indicated that antibiotic concentrations, metal concentrations, bacterial community composition, and MGE composition were not significantly associated with ARG, MRG, or BRG profiles in either fertilized or control soils (*p* > 0.05).

## 4 Discussion

### 4.1 Impact of anaerobic digestion and slurry application on antibiotic and metal concentrations

Anaerobic digestion significantly reduced residual concentrations of ampicillin, tetracycline, erythromycin, and lomefloxacin in swine manure, with removal efficiency varying by antibiotic type. Notably, ampicillin was completely removed (100%, *p* < 0.05), consistent with previous studies highlighting the effective removal of ampicillin by anaerobic digestion compared to other antibiotics (Gurmessa et al., 2020). The high removal efficiency may be attributed to the hydrolysis of *β*-lactam rings through biodegradation and the acidogenesis stage of anaerobic digestion that inactivate ampicillin (Mitchell et al., 2013; Lima et al., 2020). Ciprofloxacin was the most abundant antibiotic, owing to its extensive use in livestock farming (Yao et al., 2024), but exhibited limited removal during anaerobic digestion. Its persistence stems from its low biodegradability and capacity for solid particle adsorption, resulting in substantial concentrations in digestates and sustained moderate resistance risks (Girardi et al., 2011). These results confirm that antibiotic type is a key factor that influences removal efficiency during anaerobic digestion.

Furthermore, the long-term application of slurries significantly increases both the prevalence and concentrations of antibiotics in soils, primarily due to incomplete removal of antibiotics during anaerobic digestion, consistent with previous studies (Zeng et al., 2024). In this study, resistance and toxicological risk levels were higher in slurry-fertilized soils than in slurries themselves. This phenomenon is attributed to the capacity of soils to absorb antibiotics through cation exchange, hydrophobic interactions, surface complexation, and cation bridging (Nkoh et al., 2024). Prolonged slurry application not only continuously introduces antibiotics into soils, but also facilitates their accumulation. Consequently, antibiotic concentrations in soils can surpass those in the applied slurries, posing heightened risks to soil microbiomes and overall environmental health.

When considering metals, anaerobic digestion significantly reduces metal concentrations and their ecological risks in swine manure, primarily due to dilution and the volatilization of certain metals like arsenic (Wu, 2024). Therefore, and in stark contrast to antibiotics, slurry application had a limited impact on soil metal content and associated environmental risks. This key difference suggests that anaerobic digestion is more effective at mitigating metal-related ecologic risks from manure than antibiotic-related risks.

### 4.2 Impact of anaerobic digestion and slurry application on ARG composition

Contrary to previous findings reporting anaerobic digestion can effectively remove ARGs from swine manure (Pu et al., 2018), this study revealed limited impacts of anaerobic digestion on the total abundance and diversity of ARGs, perhaps due to differences in anaerobic digestion technologies (Zhang M. et al., 2021). Moreover, even the modularity of ARG networks increased during anaerobic digestion, suggesting the formation of stable and interconnected ARG network structures. Enhanced modularity may contribute to ARG resilience, rendering them less susceptible to removal (de Vries et al., 2018). These results suggest that interactions among ARGs are primarily responsible for their persistence and resistance to removal during anaerobic digestion.

Subsequently, with the application of slurry to soils, the long-term application of swine slurry significantly increased the total abundance and diversity of ARGs in soils, consistent with previous studies (Zeng et al., 2024). A notable finding was that approximately 78.2% of ARG subtypes in fertilized soils were shared with slurry samples, while no ARGs were uniquely shared between slurry and control soil samples. This pattern indicates that ARG dissemination is primarily attributable to the direct introduction of exogenous genes from slurry rather than independent selection in unamended soils (Zeng et al., 2024). Moreover, residual antibiotics and heavy metals in the slurry likely exerted selective pressures that facilitated the proliferation and emergence of novel ARGs within resident soil microbial communities (Liu et al., 2024). Collectively, these results provide robust evidence that swine slurry serves as a major vector for the introduction and propagation of ARGs in agricultural environments.

The low abundance of ARGs observed in our control soils aligns with studies focusing on pristine forest ecosystems (Song et al., 2021). These undisturbed environments typically exhibit antibiotic concentrations below detectable levels, resulting in minimal selective pressure for ARG enrichment (Song et al., 2021; Peng et al., 2022). Moreover, although ARGs are not entirely absent in pristine settings, their limited association with MGEs suggests a low potential for horizontal gene transfer, which contributes to their overall low prevalence and reduced environmental risk (Zhang et al., 2024). National-scale surveys have confirmed that even undisturbed forest soils across China harbor ARGs, but their relative abundances remain consistently low (e.g., ranging from 6.20 × 10^−7^ to 2.52 × 10^−3^ copies/16S rRNA), significantly lower than those found in soils impacted by human activity, such as agricultural amendments (Pu et al., 2018; Song et al., 2021). For example, Lemos et al. reported abundant and diverse ARG reservoirs in Amazonian forest soils that were affected by deforestation, a well-documented driver of increased ARG abundance and diversity (Lemos et al., 2021). These findings provide compelling evidence that human activity is a critical factor influencing the soil resistomes (Wu et al., 2023).

### 4.3 Impact of anaerobic digestion and slurry application on co-occurrence of ARGs with MRGs and MGEs

To understand the mechanisms behind ARG persistence and spread, we investigated their co-occurrence with other genetic elements. Although the co-occurrence frequency of ARGs and MRGs decreased during anaerobic digestion, alongside reduced MRG abundances, it is important to note that co-occurrence of the multidrug resistance gene *mdtL* and the chromium resistance gene *yieF* persisted in slurry samples. The co-selection of ARGs and MRGs is a key mechanism by which metals influence ARG prevalence (Li X. et al., 2022). Coincidently, and supporting this idea, chromium concentrations were found had significantly association with ARG variation in both manure and slurry samples (*p* < 0.05). By comparison, significant associations were not observed between antibiotics and ARGs (*p* > 0.05). These results are consistent with those showing stronger associations between metals and ARGs compared to antibiotics (Li X. et al., 2022). Therefore, greater attention should be directed toward addressing metal-driven co-selection of ARGs to mitigate associated environmental risks.

Beyond metal co-selection, the role of MGEs proved to be crucial (Zeng et al., 2024). Anaerobic digestion increased prevalence of ARG-MGE co-occurrence patterns, numbers of potential mobile ARG-carrying contigs, and greater diversity of potentially mobile ARG types in slurries, alongside increased MGE diversity. This suggests that the digestion process may inadvertently promote the genetic mobility of ARGs (Zhang M. et al., 2021). Specifically, MGEs were a major factor governing the composition of ARGs in slurry, but significant associations were not observed between MGEs and ARGs in swine manure samples (*p* > 0.05). We hypothesize that higher fluidity in slurries compared to solid manure likely facilitated the diffusion of ARGs and MGEs, increasing their contact frequency and enhancing the influence of MGEs on ARGs, leading to enhanced ARG mobility during anaerobic digestion (Wang et al., 2024a). Furthermore, the co-occurrence of ARGs and MGEs in soils is also enhanced by long-term slurry application that facilitates HGT of ARGs between microorganisms and plays a key role in observed increases in soil ARGs (Liu et al., 2020).

Direct evidence for HGT was observed through high sequence homology of ARGs (including *tet(M)*, *APH(4)-Ia*, *AAC(3)-IV*, and *tet(A)*) across different bacterial species during anaerobic digestion. More diverse bacterial hosts of ARGs were identified in the slurry samples, further suggesting HGT of ARGs between different bacterial species during anaerobic digestion (Pan et al., 2024). HGT of these potentially mobile ARGs was inferred between *E. coli* and other pathogenic bacteria, likely due to the large and open genome of *E. coli* that facilitates the integration of exogenous ARGs through HGT (Zhao et al., 2020; Pan et al., 2022; Ullah et al., 2023). Of particular concern, seven potentially mobile ARGs were also likely transferred between *E. coli* strains during anaerobic digestion. Among these, *tet(M)* and *tet(A)* were inferred to have been transferred among *E. coli* strains and between *E. coli* and other pathogens including *E. faecalis* and *S. enterica*. These results are consistent with previous studies documenting plasmid-mediated transfer of *tet(X4)* from *E. coli* LHM10-1 to *E. coli* C600, *S. enterica*, and *K. pneumoniae* through conjugation assays (Sun et al., 2019). The significantly higher relative abundance of *tet(M)* in the slurry samples compared to the manure samples further supports the hypothesis that the widespread distribution of certain ARGs may be primarily attributed to their HGT capacities (Wang et al., 2024a).

Critically, the potential for HGT extends beyond the digester into the environment. Eight potentially mobile ARGs were identified as likely undergoing HGT between slurry-derived *E. coli* strains and soil-derived *E. coli* strains. Similarly, the combination of *sul2* and *IS91* family transposases was detected in both manure and manure-amended soils, but were annotated to different bacterial hosts, further indicating the potential for HGT in manure-amended soils (Zhang H. et al., 2020). Thus, in addition to promoting the spread of slurry-derived ARGs into soils, HGT of ARGs also prolongs the persistence of certain ARGs in soils (Li X. et al., 2022). Therefore, effective mitigation of ARGs during anaerobic digestion requires targeted reduction of MGE abundance and diversity, coupled with interventions to minimize MGE-ARG interactions. This optimization strategy is critical for controlling AMR dissemination from swine manure agricultural applications.

### 4.4 Impact of anaerobic digestion and slurry application on pathogenic host and health risks of ARGs

The dissemination risk of ARGs is ultimately tied to their bacterial hosts (Venglovsky et al., 2009). Bacterial communities and ARGs exhibited significant associations in both manure and slurry samples (*p* < 0.05), consistent with previous studies (Liu et al., 2021). These patterns have also been observed in studies of humans, livestock manure, sludge, and soils (Han et al., 2018; Zhou et al., 2018; Liu et al., 2024). *E. coli* is a common ARG host in humans, livestock, and environmental samples that poses significant health risks (Venglovsky et al., 2009; Delgado-Blas et al., 2021). In our study, *E. coli* was the predominant ARG host in both swine manure and slurry samples in this study, primarily carrying multidrug resistance genes. However, a key finding was that the relative abundances of *E. coli* significantly decreased during anaerobic digestion (*p* < 0.05), consistent with previous investigations of swine wastewater treatment (Zhang M. et al., 2021). This reduction is likely attributed to its inhibited growth during acidogenesis and methanogenesis (Tang et al., 2024). Concurrently, the abundances of multi-drug resistance genes during anaerobic digestion also decreased (*p* < 0.05). In contrast, the abundances of aminoglycoside, tetracycline, and MLS resistance genes significantly increased during anaerobic digestion (*p* < 0.05). These results can be attributed to their prevalence not only in *E. coli* but also in *S. suis*, a zoonotic pathogen whose relative abundances significantly increased during digestion (*p* < 0.05) (Weinert et al., 2015). The consistent correlation between ARG abundances and their primary host abundances suggests that improving the removal efficiency of key bacterial ARG hosts during anaerobic digestion is an effective strategy for controlling ARG proliferation.

The application of slurry to soils significantly alters the pathogenic landscape and associated risks. It significantly increases the prevalence of pathogens, while the rich nutrient content of slurries stimulates the proliferation of native pathogens, thereby increasing their abundances and ARG encoding capacity (Zhang et al., 2024). In our fertilized soils, *E. coli* was the dominant pathogen in fertilized soils and the primary host of ARGs in this study, consistent with previous investigations (Zhang S. et al., 2020). The HGT of ARGs among *E. coli* strains, as previously discussed, contributes to their development of multidrug resistance, poses a significant challenge to improving clinical efficacy of antibiotics, enhances bacterial adaptability, and facilitates their long-term colonization of soils (Li et al., 2017; Moura De Sousa et al., 2023). Given the critical role of *E. coli* in carrying and transferring ARGs, alongside its significant impact on human health, we propose that it can serve as a biomarker for assessing health risks associated with the application of swine manure in land.

Although ARGs are environmentally ubiquitous, most do not pose significant public health risks unless they are carried by pathogens and co-localized with MGEs (Oh et al., 2018). Thus, to assess the health implications, moving beyond mere quantification of high-risk ARG abundance is necessary. Our health risk analysis revealed that, unlike the invariability of total ARG abundances, the total abundance of Rank I ARGs significantly increased after anaerobic digestion (*p* < 0.05). Consistent with changes in Rank I ARGs, the ARG risk index also significantly increased after anaerobic digestion (*p* < 0.05). Similarly, Liu *et al.* reported that anaerobic digestion did not consistently reduce the risks of sludges due to the elevated proportion of Rank I ARGs after treatment (Liu et al., 2024). Moreover, the use of swine slurries in soils led to a nearly 96.7% increase in new Rank I ARGs. Concurrently, slurry application significantly increased the ARG risk index for soils (*p* < 0.05). This consistency indicates that changes in Rank I ARGs can, to some extent, reflect variation in the ARG risk index. Similarly, Liao *et al.* reported gradual increases in human-associated ARGs in association with higher dosages of sewage sludge and manure fertilizer (Liao et al., 2022). Consequently, we argue that the diversity and abundance of ARGs alone are insufficient to assess their health risks. Rather, evaluating the variation of Rank I ARGs and calculating the ARG risk index are necessary steps to understand the health risks associated with ARGs in swine manure following anaerobic digestion and soil application. Collectively, these findings underscore the necessity of enhancing anaerobic digestion to preferentially remove mobile and high-risk ARGs, thereby mitigating the dissemination of ARG-associated health risks from agricultural swine manure application.

This study offers valuable insights into resistomes dynamics and health risks associated with anaerobic digestion and slurry application, yet several limitations must be acknowledged. The findings are constrained by the study’s geographical scope (limited to Sichuan Province), temporal design (single time-point sampling), and operational focus (one common anaerobic digestion paradigm) (Zhang M. et al., 2021; Guruge et al., 2025). As a result, the effects of regional differences, seasonal variations, and diverse technological approaches on the resistomes remain unassessed. Future studies incorporating expanded sampling across multiple regions, seasons, and diverse operational systems would be invaluable for obtaining a more systematic and comprehensive understanding of the factors governing resistomes dynamics and associated health risks, thereby enabling more accurate guidance for optimizing anaerobic digestion processes.

A further challenge lies in the practical difficulty of obtaining true baseline soil samples prior to historical fertilization, compounded by confounding factors such as past land use and rainfall-mediated dispersal that may compromise control soil integrity. Although a chronosequence approach and comparison with forest soil were employed as environmental references, these serve as indirect proxies rather than direct longitudinal measures (Huggett, 1998). To more accurately the effects of slurry application, future studies should establish well-isolated experimental plots with documented amendment histories, enabling collection of true baseline samples and systematic time-series monitoring after slurry application.

## 5 Conclusion

Comprehensive evaluation of the variation and risks of antibiotics, metals, ARGs, MRGs, and MGEs were conducted in this study for swine manure during anaerobic digestion and subsequent soil application of anaerobic slurries. While anaerobic digestion significantly reduced concentrations and associated risks of most antibiotics (except ciprofloxacin) and metals, it still exacerbated related risks through soil antibiotic accumulation and persistent ARG-MRG co-selection in slurries. Furthermore, the treatment demonstrated limited effectiveness in reducing total ARG abundance and even increased specific high-risk ARG variants, consequently elevating ARG-related health risks in fertilized soils. These phenomena were mediated by complex interactions between ARGs, bacterial communities, and MGEs that collectively maintain and amplify ARG prevalence from manure to soil. These findings highlight the critical need to optimize anaerobic digestion processes to specifically target MGE reduction and limit pathogenic host proliferation, while simultaneously developing science-based guidelines for long-term digestate application to protect soil ecosystem health.

## Data Availability

The raw sequence data have been deposited in the Genome Sequence Archive (GSA) at the China National Center for Bioinformation (CNCB-NGDC) with the accession numbers CRA049976, CRA049978, and CRA049837.
