# Supplementary material for: Variation and spread of resistomes in swine manure, manure slurries, and long-term manure-fertilized soils
Source: Front Microbiol. 2025 Oct 29;16:1683394. doi: 10.3389/fmicb.2025.1683394 (PMC12605092; doi:10.3389/fmicb.2025.1683394)
Supplement: Supplementary file 1 [file Data_Sheet_1.docx]

Supporting Information (SI) for

**Variation and spread of resistomes in swine manure, manure slurries, and long-term manure-fertilized soils**

**Supplementary Figures**


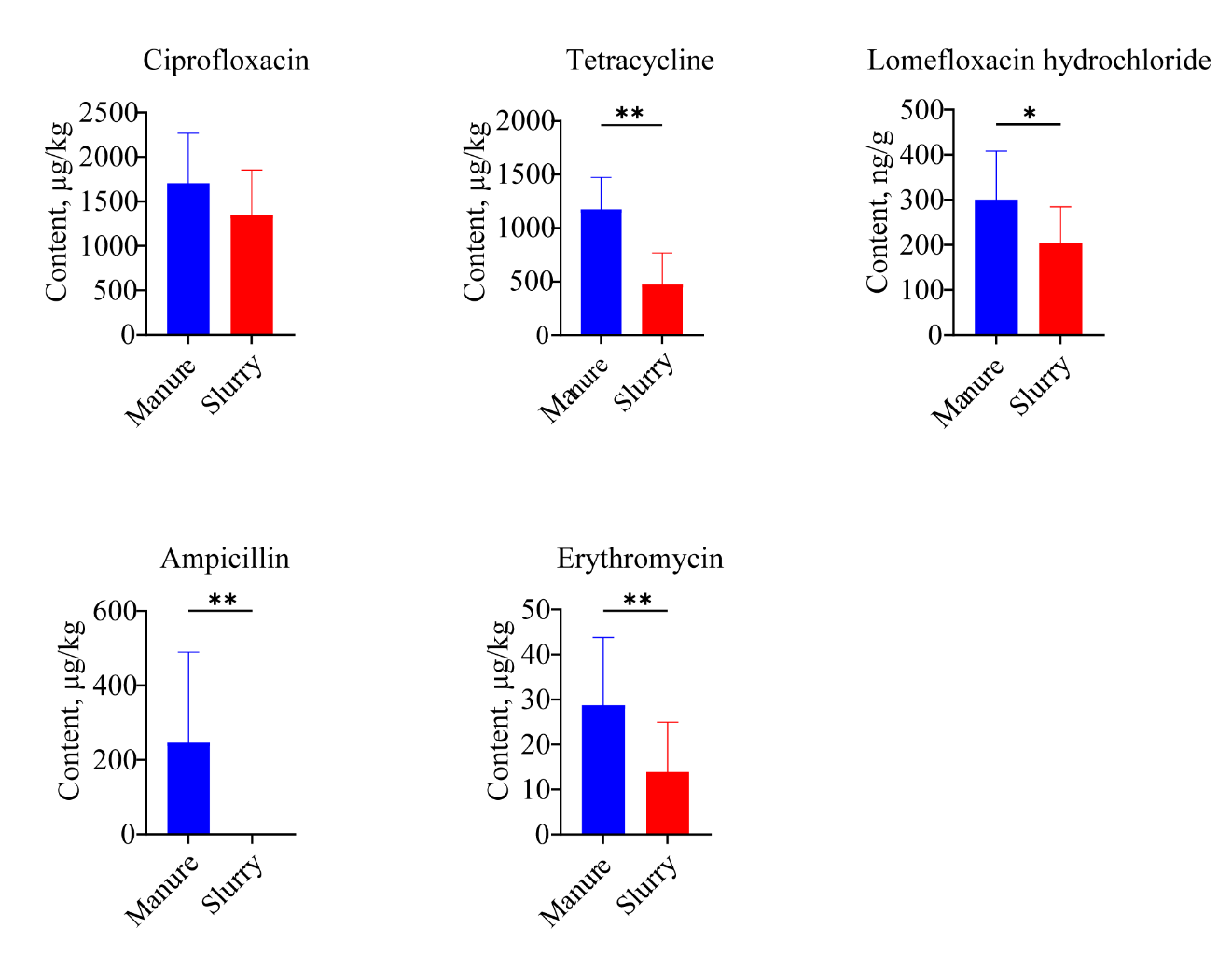


**Figure S1. Concentrations of antibiotics in swine manure and slurries.** *: *p* < 0.05; **: *p* < 0.01.

**
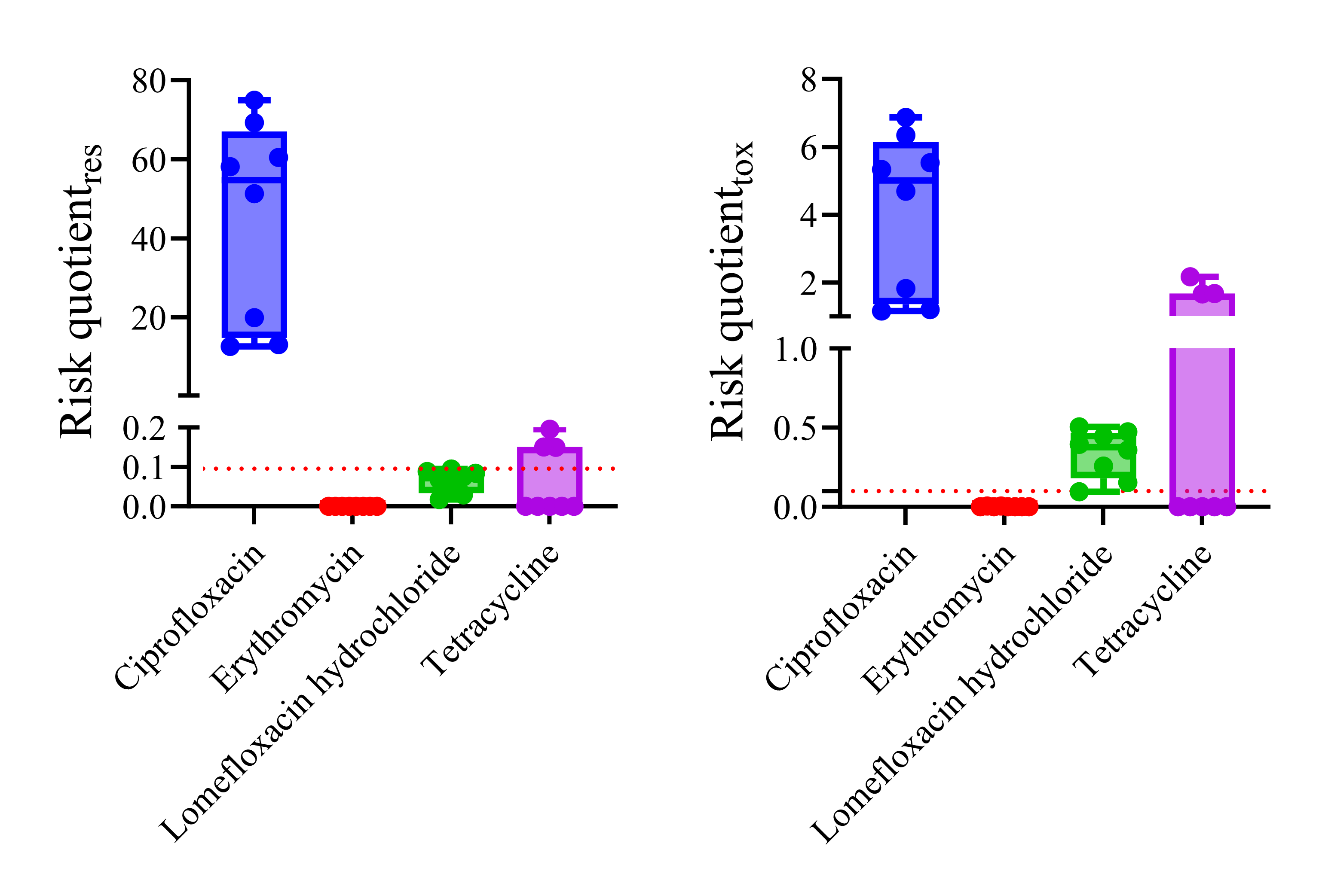
 Figure S2. The resistance and toxicological risks of antibiotics in fertilized soils.** Red dotted line indicates the 0.1 threshold. An RQ < 0.1 indicates low risk; 0.1 ≤ RQ ≤ 1 indicates moderate risk; RQ > 1 indicates high risk.


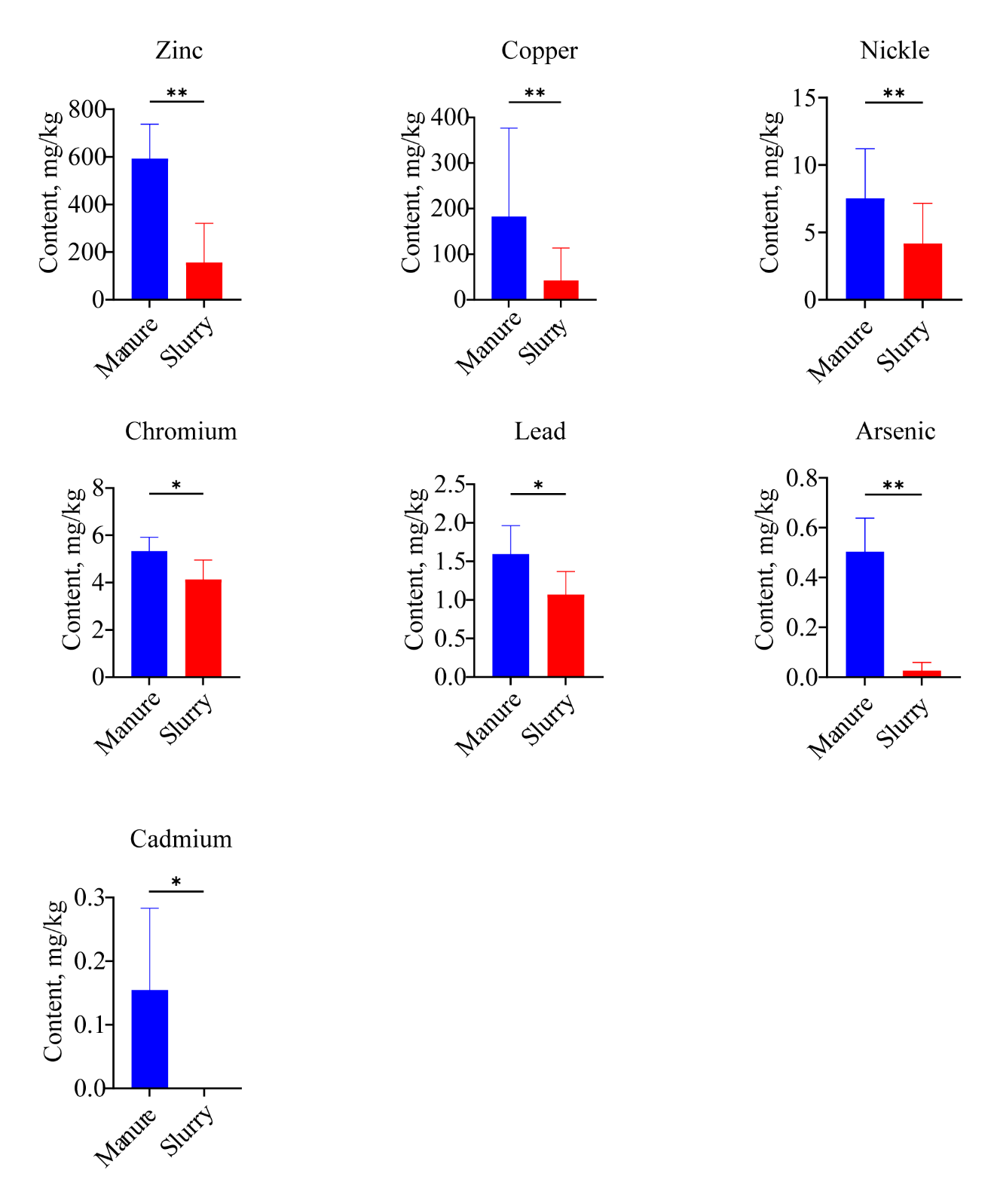


**Figure S3. Concentrations of metals in swine manure and slurries.** *: *p* < 0.05; **: *p* < 0.01.


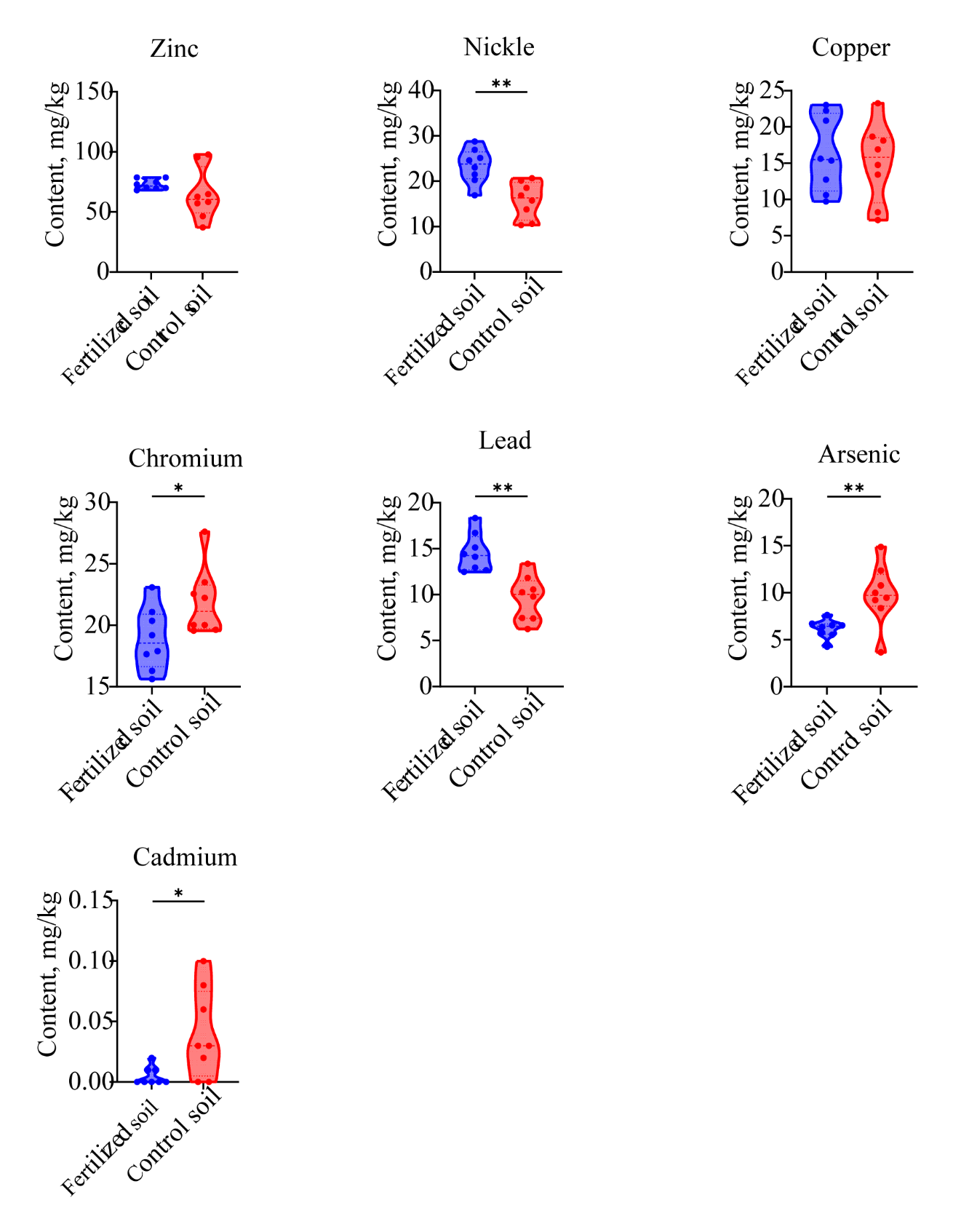
 **Figure S4. Concentrations of metals in fertilized and control soils.** *: *p* < 0.05; **: *p* < 0.01.


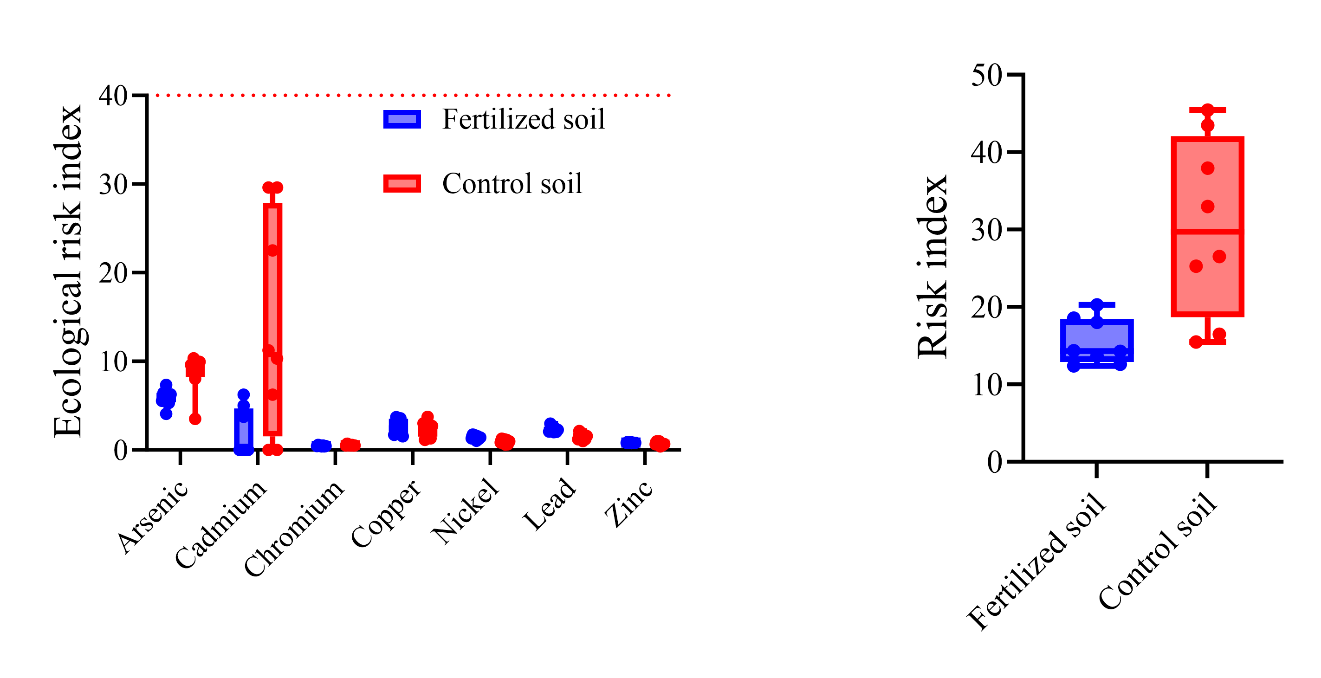


**Figure S5. Ecological risk index and risk index values for metals in fertilized and control soils.** Red dotted line indicates the 40 thresholds.

**Figure S6. The composition of antibiotic resistance genes (ARGs) in swine manure and slurry samples.**

**
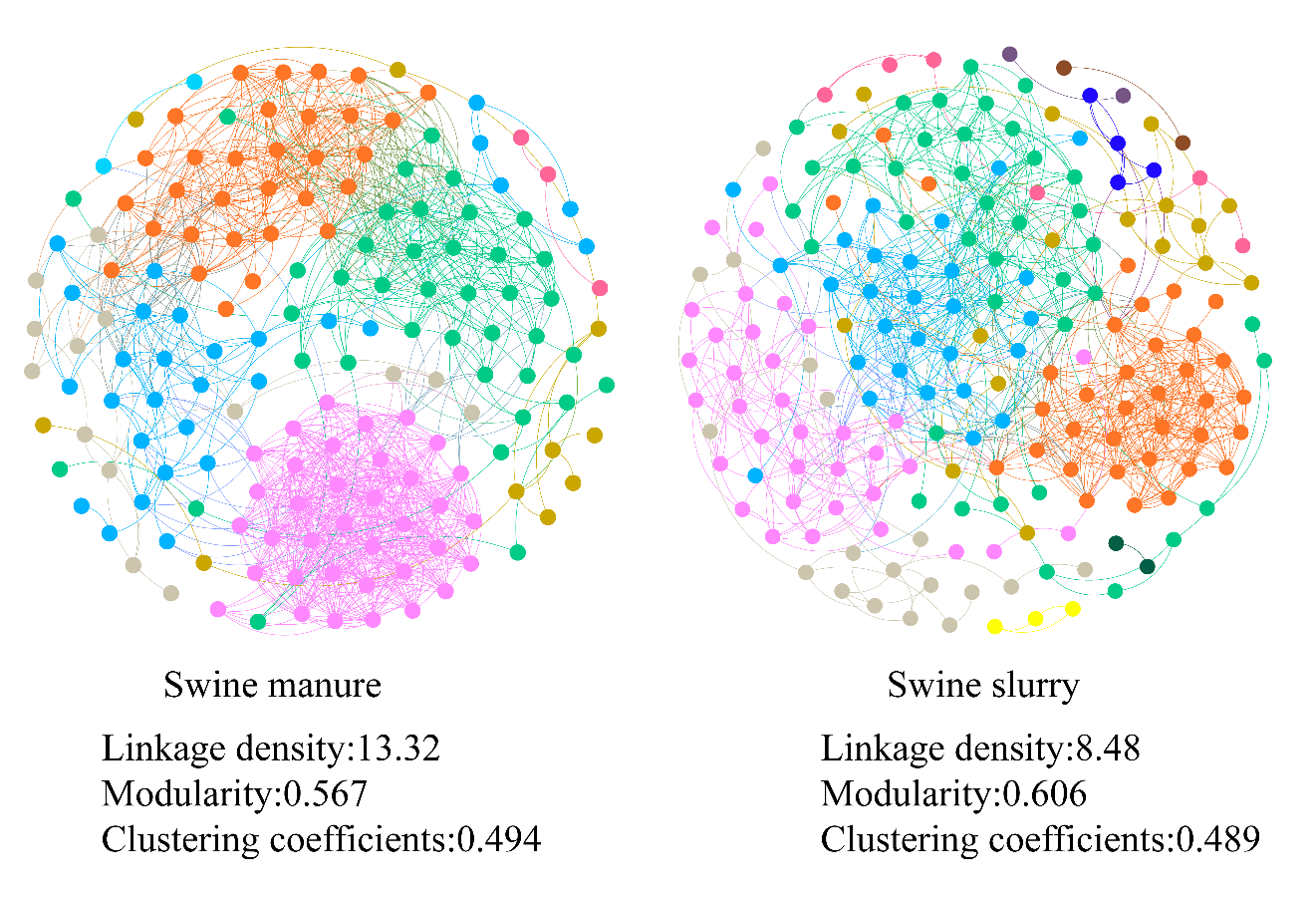
**

**Figure S7. Co-occurrence network of antibiotic resistance genes (ARGs) in swine manure and slurry samples, based on significant correlations in abundances (Spearman's ρ > 0.6, *p* < 0.01).** Nodes are colored by modularity class that were identified through network analysis.

**Figure S8. The composition of antibiotic resistance genes (ARGs) in fertilized and control soils.**


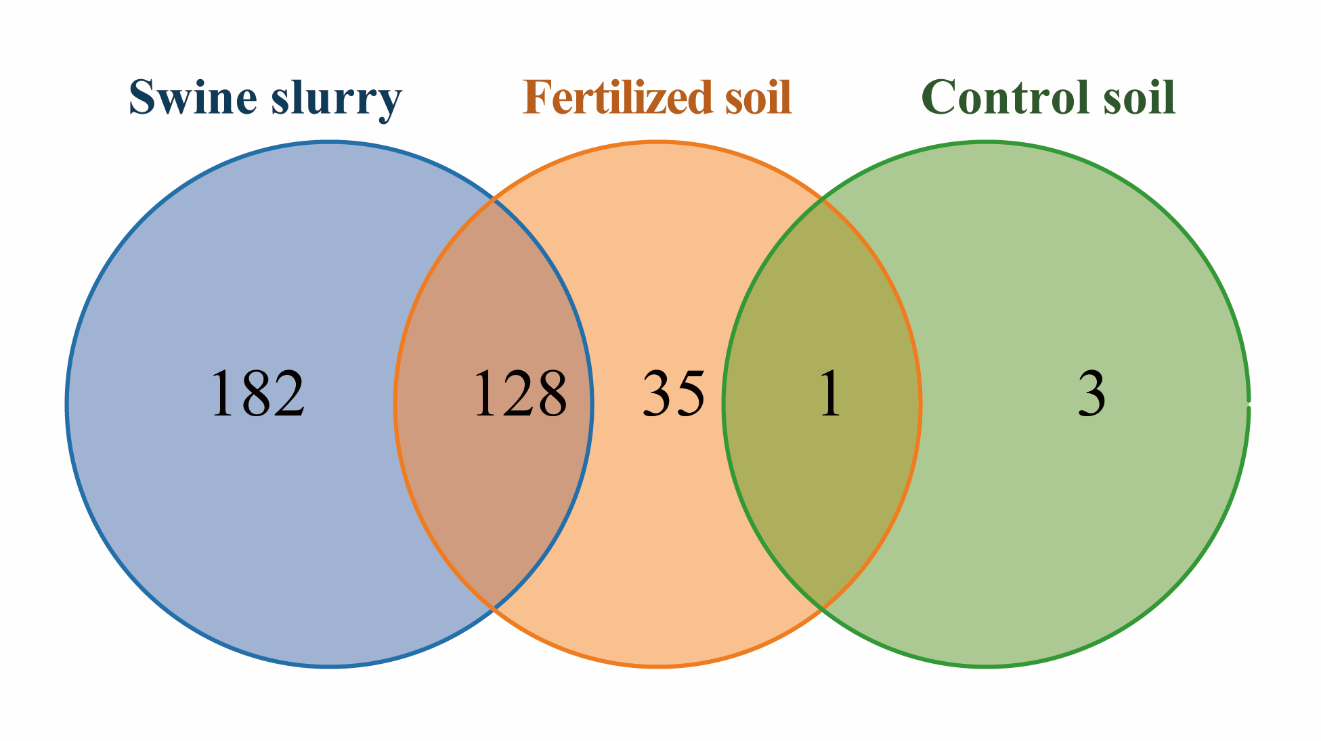


**Figure S9. Venn diagram showing sharing of antibiotic resistance genes (ARGs) among swine slurry, fertilized soil, and control soil samples.**

**Figure S10. The composition of metal resistance genes (MRGs) in swine manure, slurry and soil samples.**

**Figure S11. The composition of mobile genomic elements (MGEs) in swine manure, slurry and soil samples.**


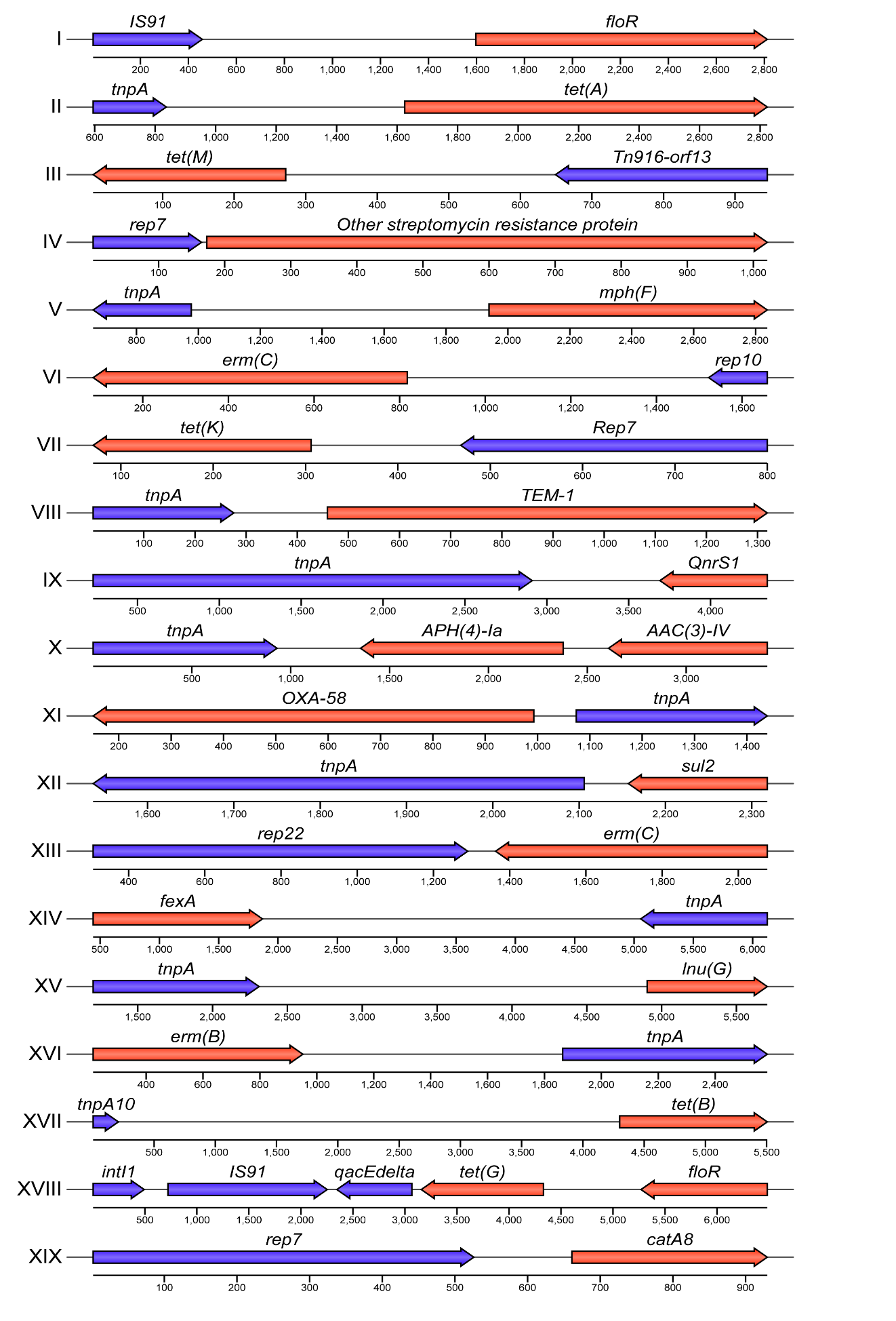


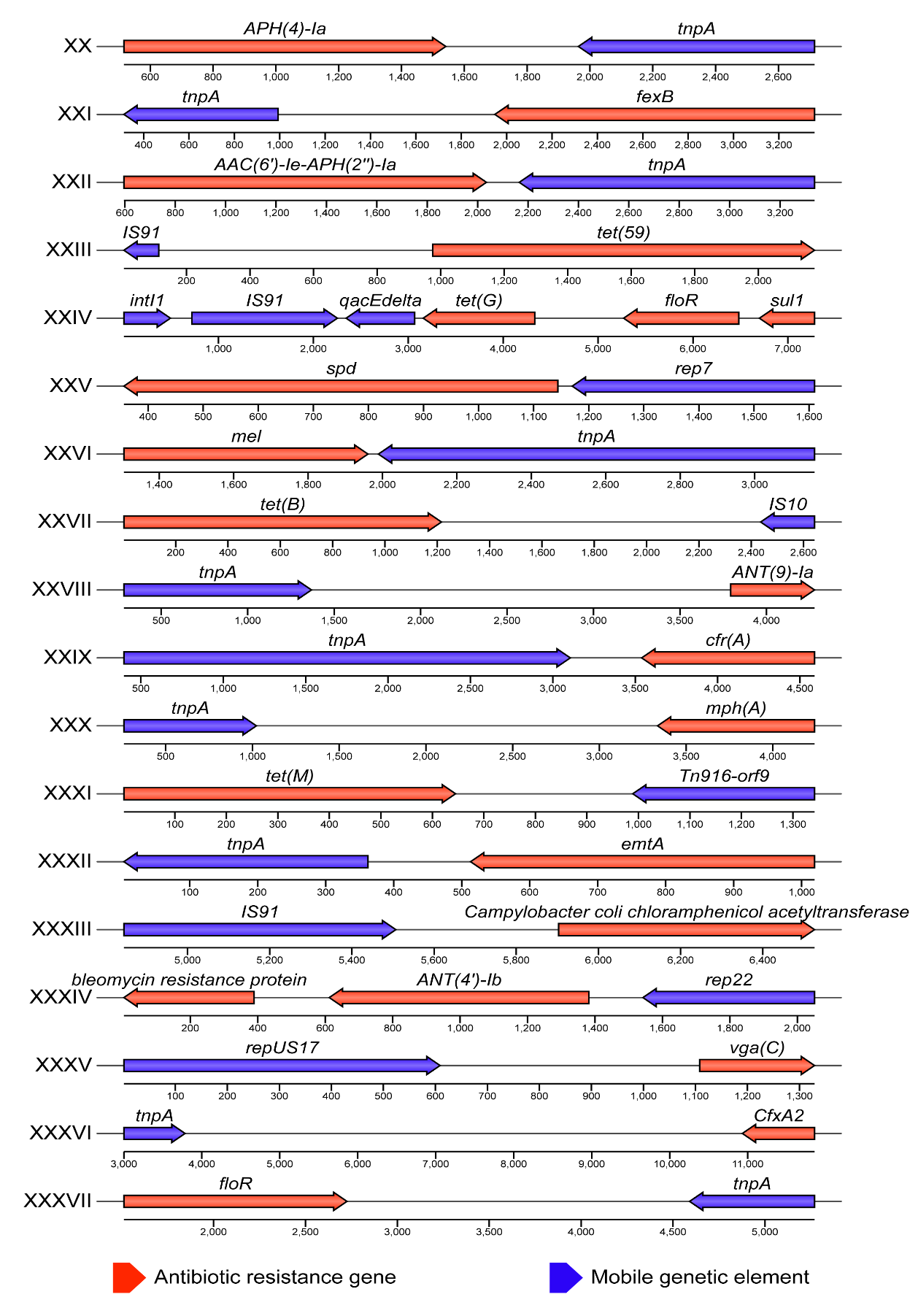


**Figure S12. The arrangement of ARGs and MGEs within co-occurrences encoding representative contigs. Co-occurrences are shared across two or more contigs.**


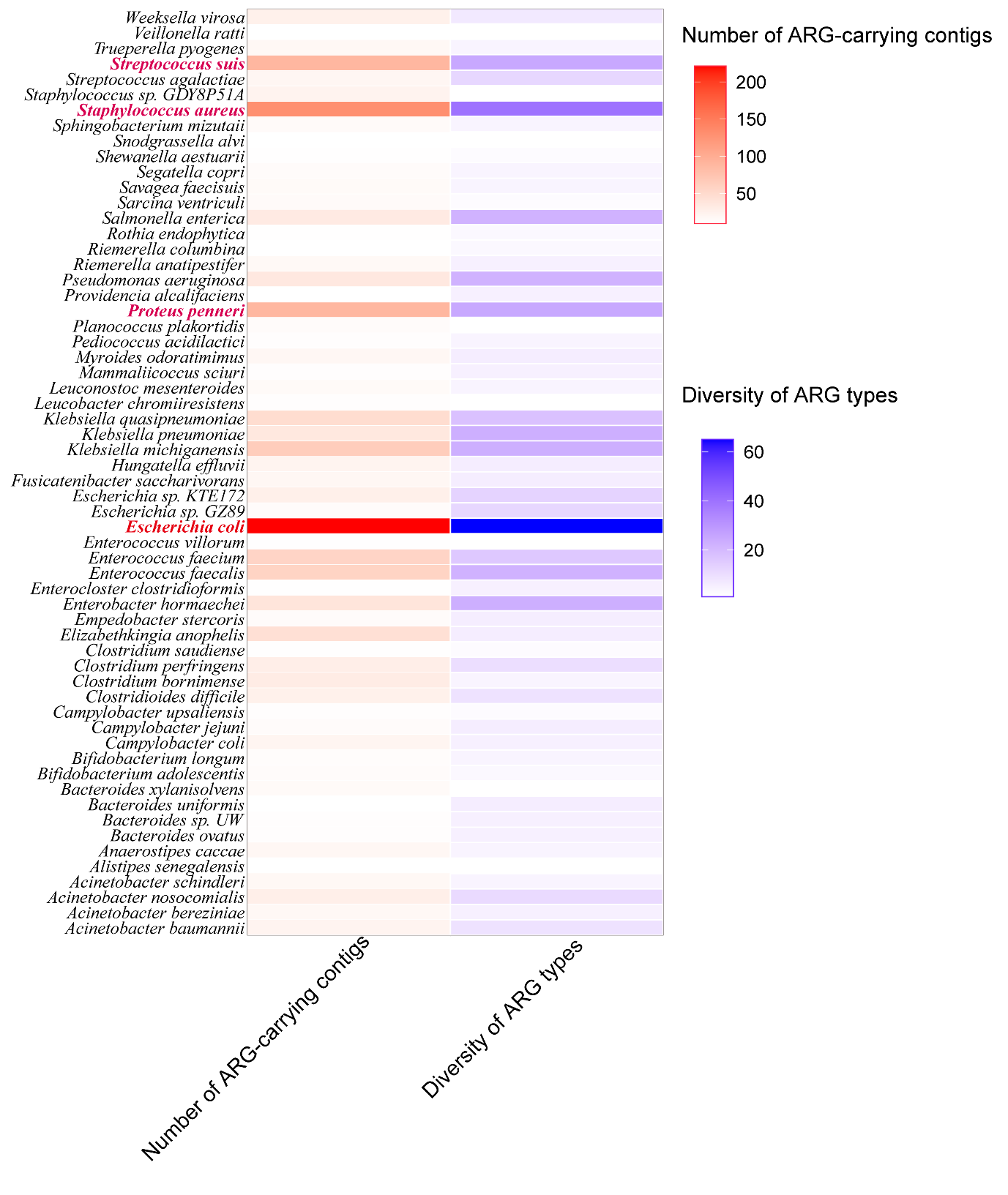


**Figure S13. Prevalence of antibiotic resistance genes (ARGs) in dominant host species identified from at least 10 ARG-carrying contigs.**

**Figure S14. Taxonomic composition of pathogenic bacterial communities in swine manure, slurry, and soil samples.**

**
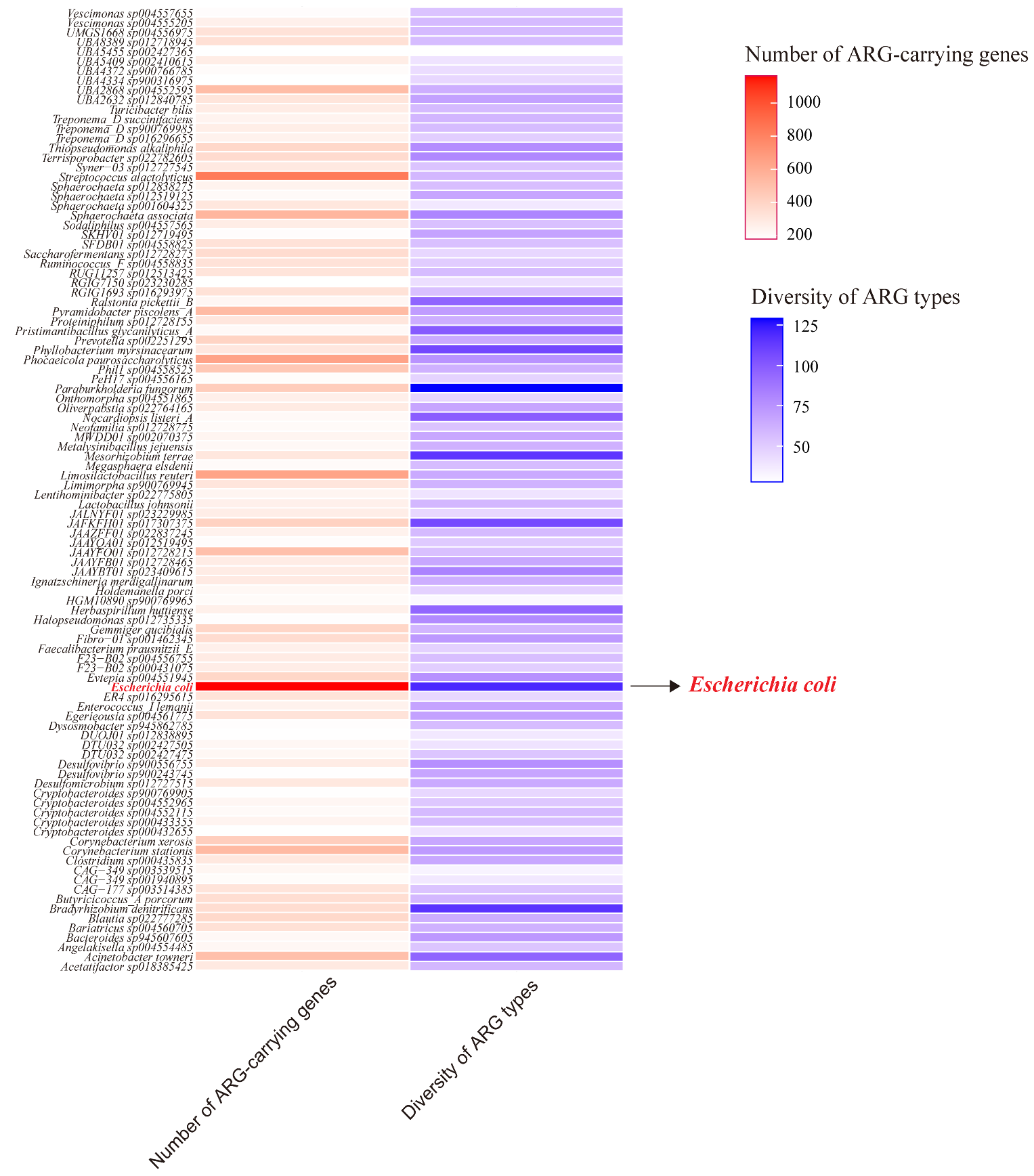
**

**Figure S15. Distribution of antibiotic resistance genes (ARGs) in the 100 metagenome-assembled genomes (MAGs) with the most ARG gene counts.**

**
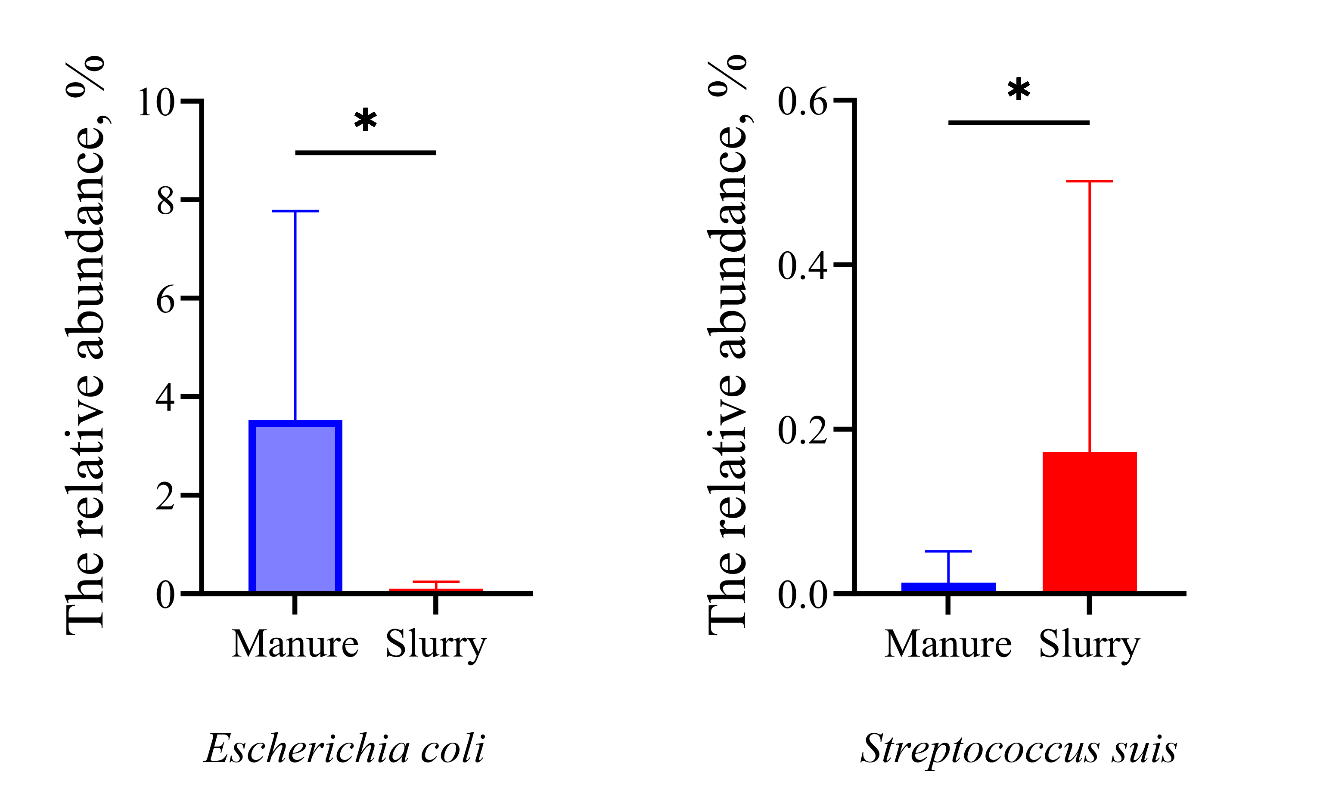
**

**Figure S16. Relative abundances of *Escherichia coli* and *Streptococcus suis* in swine manure and slurry samples.**

**Figure S17. Type of high-risk antibiotic resistance genes (ARGs) in swine manure, slurry, and soil samples.**

**
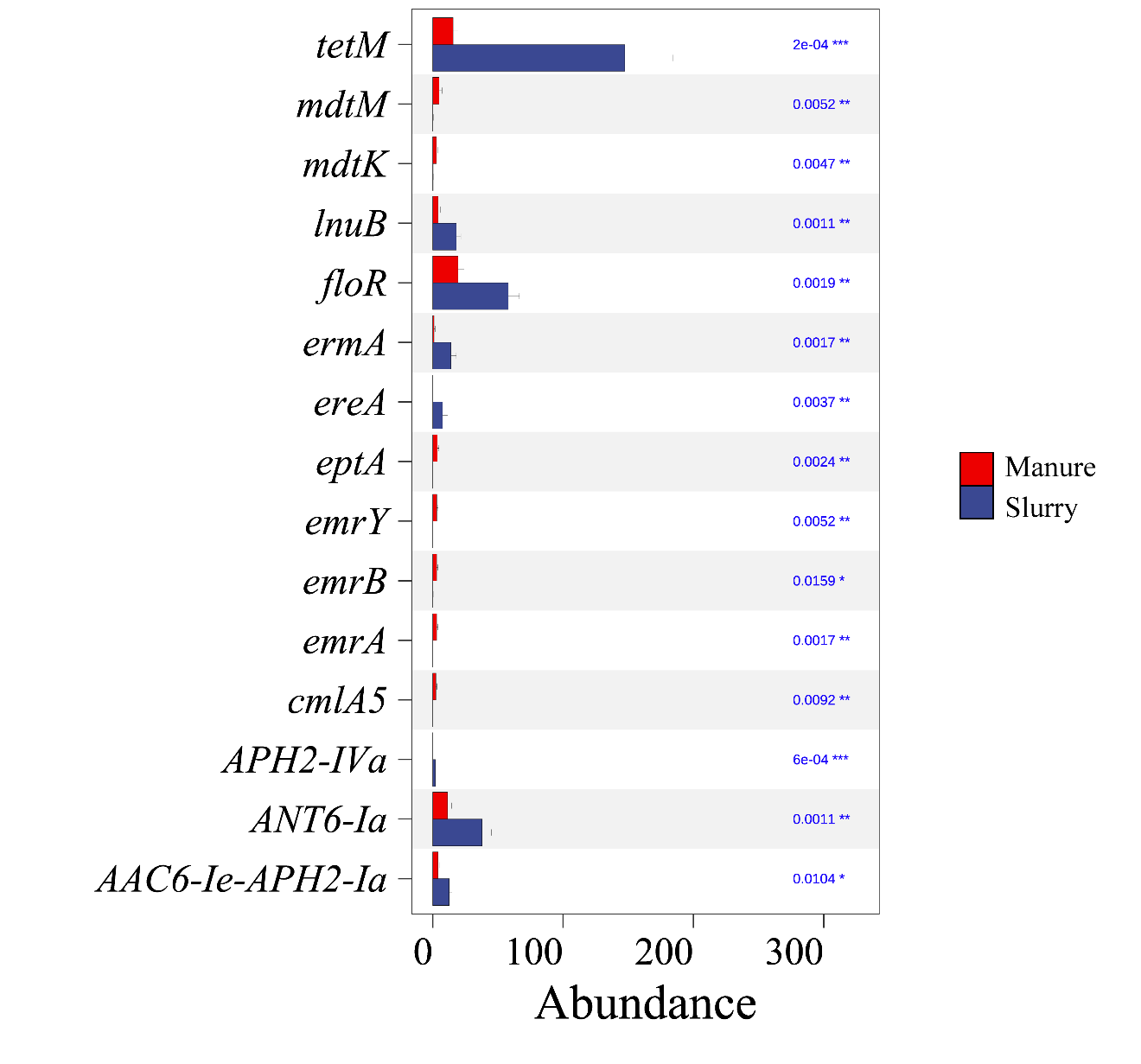
**

**Figure S18. Significantly different high-risk antibiotic resistance genes (ARGs) in swine manure and slurry samples (*p* < 0.05).**

**
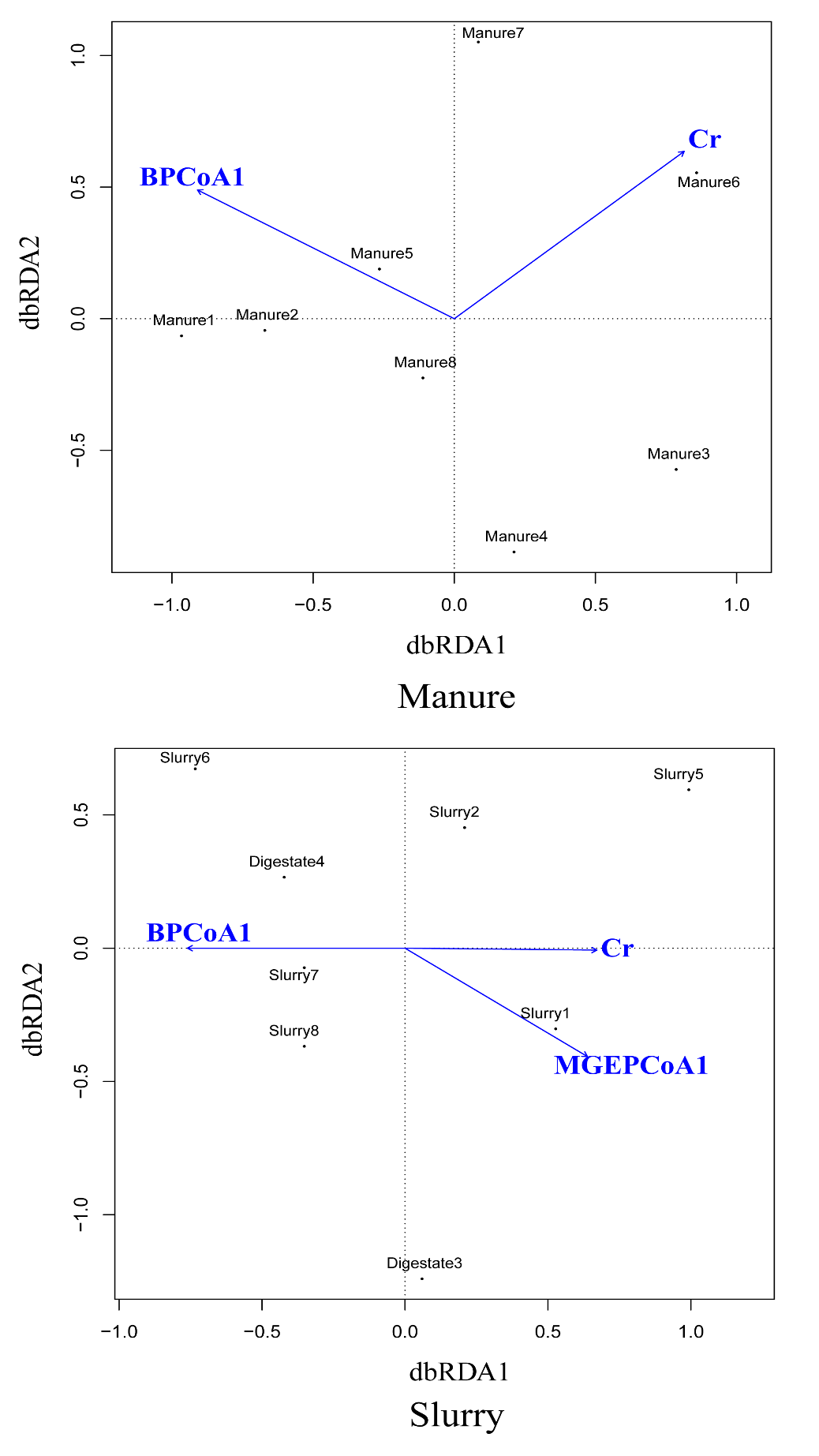
**

**Figure S19. Distance-based redundancy analysis (dbRDA) of antibiotic resistance gene (ARGs) profiles in swine manure and slurry samples based on permutational multivariate analysis of variance (PERMANOVA) tests.**

**Supplementary Table**

**Table S1. The content of antibiotics in swine manure, slurry and soil.**

| Sample style | Sample | Ampicillin  (µg/kg) | Ciprofloxacin  (µg/kg) | Erythromycin  (µg/kg) | Lomefloxacin hydrochloride  (µg/kg) | Tetracycline  (µg/kg) |
| --- | --- | --- | --- | --- | --- | --- |
| Swine manure | Manure1 | 175.18 | 1475.26 | 25.41 | 456.60 | 1658.51 |
|  | Manure2 | 199.80 | 1551.69 | 7.6 | 315.31 | 988.62 |
|  | Manure3 | 180.56 | 1409.46 | 16.85 | 287.36 | 749.40 |
|  | Manure4 | 300.60 | 730.48 | 17.51 | 113.39 | 1160.24 |
|  | Manure5 | 151.55 | 1606.68 | 30.17 | 251.94 | 1049.74 |
|  | Manure6 | 12.82 | 2102.15 | 34.59 | 370.06 | 1530.85 |
|  | Manure7 | 133.92 | 2454.21 | 46.83 | 219.70 | 1229.22 |
|  | Manure8 | 816.07 | 2315.04 | 51.08 | 388.23 | 1042.80 |
|  | Total | 1970.51 | 13644.97 | 230.04 | 2402.58 | 9409.38 |
|  | Mean | 246.31 | 1705.62 | 25.57 | 300.32 | 1176.17 |
|  | Max | 816.07 | 2454.21 | 51.08 | 456.60 | 1658.51 |
|  | Min | 12.82 | 730.48 | 7.60 | 113.39 | 749.40 |
|  | DR | 100.00% | 100.00% | 100.00% | 100.00% | 100.00% |
|  | SD | 227.83 | 526.09 | 14.09 | 100.74 | 276.72 |
| Swine slurry | Slurry1 | — | 1419.42 | 17.80 | 300.69 | 865.36 |
|  | Slurry2 | — | 1332.77 | ND | 213.95 | 401.74 |
|  | Slurry3 | — | 1571.71 | ND | 240.46 | 248.29 |
|  | Slurry4 | — | 268.99 | 10.48 | 67.73 | 169.95 |
|  | Slurry5 | — | 1537.13 | 16.04 | 223.09 | 429.60 |
|  | Slurry6 | — | 1923.79 | 27.44 | 291.89 | 904.28 |
|  | Slurry7 | — | 1681.19 | 29.52 | 173.14 | 163.15 |
|  | Slurry8 | — | 1030.97 | 9.84 | 115.56 | 610.92 |
|  | Total | — | 10765.97 | 111.11 | 1626.51 | 3793.28 |
|  | Mean | — | 1345.75 | 20.25 | 203.31 | 474.16 |
|  | Max | — | 1923.79 | 29.52 | 300.69 | 904.28 |
|  | Min | — | 268.99 | 9.84 | 67.73 | 163.15 |
|  | DR | 0.00% | 100.00% | 0.75% | 100.00% | 100.00% |
|  | SD | — | 474.13 | 7.61 | 76.00 | 274.83 |
| Fertilized soil | Fertilized soil1 | — | 532.60 | — | 106.56 | 213.69 |
|  | Fertilized soil2 | — | 349.57 | — | 39.12 | 164.03 |
|  | Fertilized soil3 | — | 1551.58 | — | 183.78 | — |
|  | Fertilized soil4 | — | 338.71 | — | 63.94 | — |
|  | Fertilized soil5 | — | 1612.57 | 12.00 | 196.15 | — |
|  | Fertilized soil6 | — | 1999.44 | 11.05 | 164.93 | 165.17 |
|  | Fertilized soil7 | — | 1367.79 | — | 148.98 | — |
|  | Fertilized soil8 | — | 1847.89 | — | 209.23 | — |
|  | Total | — | 9600.16 | 23.05 | 1112.69 | 542.89 |
|  | Mean | — | 1200.02 | 11.52 | 139.09 | 180.96 |
|  | Max | — | 1999.44 | 12.00 | 209.23 | 213.69 |
|  | Min | — | 338.71 | 11.05 | 39.12 | 164.03 |
|  | DR | 0.00% | 100.00% | 0.25% | 100.00% | 0.38% |
|  | SD | — | 641.42 | 0.47 | 58.78 | 23.15 |
| Control soil | Control soil1 | — | — | — | — | — |
|  | Control soil2 | — | — | — | — | — |
|  | Control soil3 | — | — | — | — | — |
|  | Control soil4 | — | — | — | — | — |
|  | Control soil5 | — | — | — | — | — |
|  | Control soil6 | — | — | — | — | — |
|  | Control soil7 | — | — | — | — | — |
|  | Control soil8 | — | — | — | — | — |
|  | Total | — | — | — | — | — |
|  | Mean | — | — | — | — | — |
|  | Max | — | — | — | — | — |
|  | Min | — | — | — | — | — |
|  | DR | 0.00% | 0.00 | 0.00% | 0.00% | 0.00% |
|  | SD | — | — | — | — | — |

Note: "—" indicates values below the method detection limit (MDL). Mean values were derived only from measurements above the MDL. DR: Detection rate.

**Table S2. The content of metals in swine manure, slurry and soil.**

| Sample style | Sample | Arsenic  (mg/kg) | Cadmium  (mg/kg) | Chromium  (mg/kg) | Copper  (mg/kg) | Nickel  (mg/kg) | Lead  (mg/kg) | Zinc  (mg/kg) |
| --- | --- | --- | --- | --- | --- | --- | --- | --- |
| Swine manure | Manure1 | 0.54 | 0.10 | 4.93 | 95.20 | 5.01 | 1.74 | 597.85 |
|  | Manure2 | 0.43 | 0.10 | 4.82 | 74.56 | 4.68 | 1.89 | 576.59 |
|  | Manure3 | 0.57 | 0.25 | 5.43 | 578.34 | 13.66 | 1.67 | 714.54 |
|  | Manure4 | 0.45 | 0.35 | 5.07 | 116.18 | 5.75 | 1.22 | 606.47 |
|  | Manure5 | 0.29 | 0.05 | 4.94 | 60.59 | 4.76 | 2.09 | 470.42 |
|  | Manure6 | 0.63 | 0.31 | 6.46 | 390.53 | 10.79 | 1.31 | 868.70 |
|  | Manure7 | 0.41 | 0.05 | 5.95 | 79.76 | 4.60 | 1.81 | 481.78 |
|  | Manure8 | 0.71 | 0.03 | 5.04 | 67.16 | 11.02 | 1.03 | 428.29 |
|  | total | 4.03 | 1.23 | 42.62 | 1462.31 | 60.27 | 12.76 | 4744.64 |
|  | mean | 0.50 | 0.15 | 5.33 | 182.79 | 7.53 | 1.60 | 593.08 |
|  | min | 0.29 | 0.03 | 4.82 | 60.59 | 4.60 | 1.03 | 428.29 |
|  | max | 0.71 | 0.35 | 6.46 | 578.34 | 13.66 | 2.09 | 868.70 |
|  | SD | 0.13 | 0.13 | 0.58 | 193.60 | 3.67 | 0.37 | 144.31 |
| Swine slurry | Slurry1 | 0.02 | 0.00 | 5.34 | 6.55 | 2.12 | 0.73 | 91.09 |
|  | Slurry2 | 0.00 | 0.00 | 3.98 | 1.91 | 1.51 | 1.05 | 39.30 |
|  | Slurry3 | 0.08 | 0.00 | 3.79 | 142.43 | 8.97 | 0.81 | 72.31 |
|  | Slurry4 | 0.06 | 0.00 | 3.54 | 54.76 | 3.57 | 0.84 | 521.06 |
|  | Slurry5 | 0.00 | 0.00 | 5.33 | 2.76 | 3.67 | 1.62 | 37.74 |
|  | Slurry6 | 0.00 | 0.00 | 3.06 | 59.26 | 8.71 | 1.09 | 215.27 |
|  | Slurry7 | 0.00 | 0.00 | 4.31 | 2.73 | 1.86 | 1.07 | 52.76 |
|  | Slurry8 | 0.05 | 0.00 | 3.70 | 7.10 | 3.04 | 1.35 | 220.70 |
|  | total | 0.21 | 0.00 | 33.06 | 277.49 | 33.45 | 8.56 | 1250.23 |
|  | mean | 0.03 | 0.00 | 4.13 | 34.69 | 4.18 | 1.07 | 156.28 |
|  | min | 0.00 | 0.00 | 3.06 | 1.91 | 1.51 | 0.73 | 37.74 |
|  | max | 0.08 | 0.00 | 5.34 | 142.43 | 8.97 | 1.62 | 521.06 |
|  | SD | 0.03 | 0.00 | 0.83 | 46.48 | 2.98 | 0.30 | 164.94 |
| Fertilized soil | Fertilized soil1 | 6.54 | 0.01 | 15.62 | 10.62 | 23.01 | 14.40 | 78.67 |
|  | Fertilized soil2 | 6.52 | 0.00 | 17.64 | 9.71 | 24.57 | 12.48 | 69.64 |
|  | Fertilized soil3 | 4.25 | 0.00 | 21.08 | 22.24 | 20.24 | 12.66 | 78.72 |
|  | Fertilized soil4 | 6.36 | 0.00 | 23.08 | 15.62 | 28.76 | 15.13 | 74.75 |
|  | Fertilized soil5 | 6.71 | 0.02 | 17.89 | 15.37 | 25.12 | 14.10 | 72.88 |
|  | Fertilized soil6 | 5.76 | 0.01 | 20.35 | 23.04 | 21.48 | 18.33 | 68.05 |
|  | Fertilized soil7 | 7.63 | 0.00 | 16.29 | 12.73 | 26.91 | 12.94 | 70.26 |
|  | Fertilized soil8 | 5.53 | 0.00 | 19.20 | 20.86 | 16.88 | 16.71 | 69.90 |
|  | total | 49.30 | 0.04 | 151.13 | 130.19 | 186.96 | 116.75 | 582.87 |
|  | mean | 6.16 | 0.01 | 18.89 | 16.27 | 23.37 | 14.59 | 72.86 |
|  | min | 4.25 | 0.00 | 15.62 | 9.71 | 16.88 | 12.48 | 68.05 |
|  | max | 7.63 | 0.02 | 23.08 | 23.04 | 28.76 | 18.33 | 78.72 |
|  | SD | 1.00 | 0.01 | 2.52 | 5.23 | 3.81 | 2.07 | 4.15 |
| Control soil | Control soil1 | 9.23 | 0.08 | 19.56 | 8.23 | 10.32 | 10.56 | 37.06 |
|  | Control soil2 | 9.47 | 0.06 | 20.02 | 18.12 | 15.75 | 7.45 | 62.67 |
|  | Control soil3 | 3.65 | 0.08 | 27.60 | 7.15 | 16.86 | 6.26 | 46.47 |
|  | Control soil4 | 9.88 | 0.03 | 19.63 | 23.27 | 20.66 | 13.36 | 58.31 |
|  | Control soil5 | 8.36 | 0.03 | 22.23 | 18.65 | 20.12 | 10.26 | 64.87 |
|  | Control soil6 | 10.78 | 0.00 | 23.49 | 14.75 | 13.78 | 7.41 | 85.52 |
|  | Control soil7 | 10.35 | 0.02 | 22.56 | 16.91 | 18.51 | 9.79 | 85.76 |
|  | Control soil8 | 9.99 | 0.00 | 20.02 | 13.43 | 10.57 | 11.82 | 57.21 |
|  | total | 71.71 | 0.29 | 175.09 | 120.51 | 126.55 | 76.89 | 497.87 |
|  | mean | 8.96 | 0.04 | 21.89 | 15.06 | 15.82 | 9.61 | 62.23 |
|  | min | 3.65 | 0.00 | 19.56 | 7.15 | 10.32 | 6.26 | 37.06 |
|  | max | 10.78 | 0.08 | 27.60 | 23.27 | 20.66 | 13.36 | 85.76 |
|  | SD | 2.12 | 0.03 | 2.76 | 5.41 | 4.00 | 2.42 | 15.92 |

**Table S3. The Sichuan region's soil background criteria** (Zhao et al. 2020).

| Metal | Chromium  (mg/Kg) | Nickel  (mg/Kg) | Copper  (mg/Kg) | Zinc  (mg/Kg) | Arsenic  (mg/Kg) | Cadmium  (mg/Kg) | Lead  (mg/Kg) |
| --- | --- | --- | --- | --- | --- | --- | --- |
| Cbi (mg/Kg) | 79.00 | 32.60 | 31.10 | 86.50 | 10.40 | 0.08 | 30.90 |

**Table S4. Distribution of co-occurrence patterns between antibiotic resistance genes (ARGs) and mobile genetic elements (MGEs) in manure, slurry, and soil.**

| Co-occurrence patterns of ARGs and MGEs | Number of contigs | | | |
| --- | --- | --- | --- | --- |
|  | Manure | Slurry | Fertilized soil | Control soil |
| I | 2 | 2 | 2 | 0 |
| II | 6 | 7 | 1 | 0 |
| III | 4 | 1 | 1 | 0 |
| IV | 3 | 4 | 1 | 0 |
| V | 2 | 1 | 1 | 0 |
| VI | 1 | 2 | 1 | 0 |
| VII | 0 | 3 | 1 | 0 |
| VIII | 5 | 3 | 0 | 0 |
| IX | 5 | 1 | 0 | 0 |
| X | 4 | 6 | 0 | 0 |
| XI | 4 | 4 | 0 | 0 |
| XII | 4 | 2 | 0 | 0 |
| XIII | 3 | 4 | 0 | 0 |
| XIV | 3 | 2 | 0 | 0 |
| XV | 3 | 1 | 0 | 0 |
| XVI | 2 | 2 | 0 | 0 |
| XVII | 2 | 2 | 0 | 0 |
| XVIII | 2 | 1 | 0 | 0 |
| XIX | 2 | 1 | 0 | 0 |
| XX | 2 | 1 | 0 | 0 |
| XXI | 2 | 1 | 0 | 0 |
| XXII | 2 | 0 | 0 | 0 |
| XXIII | 1 | 3 | 0 | 0 |
| XXIV | 1 | 2 | 0 | 0 |
| XXV | 1 | 2 | 0 | 0 |
| XXVI | 1 | 2 | 0 | 0 |
| XXVII | 1 | 1 | 0 | 0 |
| XXVIII | 1 | 1 | 0 | 0 |
| XXIX | 1 | 1 | 0 | 0 |
| XXX | 1 | 1 | 0 | 0 |
| XXXI | 0 | 6 | 0 | 0 |
| XXXII | 0 | 3 | 0 | 0 |
| XXXIII | 0 | 2 | 0 | 0 |
| XXXIV | 0 | 2 | 0 | 0 |
| XXXV | 0 | 2 | 0 | 0 |
| XXXVI | 0 | 2 | 0 | 0 |
| XXXVII | 0 | 2 | 0 | 0 |

Note: Patterns labeled with Roman numerals correspond to genomic arrangements detailed in Figure S13.

**Table S5. The resistance phenotypes of *Escherichia coli*.**

| Strains | Antibiotic resistance phenotypes | Type of samples | Source of samples | Whole genome sequencing |
| --- | --- | --- | --- | --- |
| F-FYH 1-2 | TET, AMX, GEN, S3, FOS, ERY | Manure | Farm1 | √ |
| F-FYH 1-3 | TET, S3, ERY | Manure | Farm1 |  |
| F-FYH 2-2 | S3, ERY | Manure | Farm1 |  |
| F-FYH 2-3 | TET, AMX, S3, ERY | Manure | Farm1 |  |
| F-FYH 3-1 | TET, S3, ERY | Manure | Farm1 |  |
| FKJ2-1 | TET, AMX, CRO, S3, FOS, ERY | Manure | Farm3 |  |
| FKJ2-2 | TET, AMX, S3, ERY | Manure | Farm3 |  |
| FKJ2-3 | TET, AMX, CRO, GEN, S3, FOS, ERY | Manure | Farm3 |  |
| FKJ3-1 | TET, AMX, CRO, S3, FOS, ERY | Manure | Farm3 |  |
| FKJ3-3 | TET, AMX, S3, FOS, ERY | Manure | Farm3 |  |
| F-LJ 2-1 | TET, AMX, S3, FOS, ERY | Manure | Farm4 | √ |
| F-LJ 2-2 | TET, AMX, CIP, S3, ERY | Manure | Farm4 | √ |
| F-LJ 2-3 | TET, AMX, S3, ERY | Manure | Farm4 |  |
| F-LJ 3-1 | TET, AMX, S3, ERY | Manure | Farm4 |  |
| F-LJ 3-2 | TET, AMX, S3, ERY | Manure | Farm4 |  |
| F-LJ 3-3 | TET, AMX, S3, ERY | Manure | Farm4 |  |
| F-LKG 1-1 | TET, S3, ERY | Manure | Farm5 |  |
| F-LKG 1-3 | TET, S3, ERY | Manure | Farm5 |  |
| F-LKG 2-1 | TET, AMX, S3, ERY | Manure | Farm5 |  |
| F-LKG 2-2 | TET, CRO, S3, ERY | Manure | Farm5 |  |
| F-LKG 2-3 | TET, S3, ERY | Manure | Farm5 |  |
| F-LKG 3-1 | TET, AMX, GEN, CIP, S3, TIG, ERY | Manure | Farm5 | √ |
| F-LKG 3-2 | TET, AMX, GEN, CIP, S3, ERY | Manure | Farm5 |  |
| F-LKG 3-3 | TET, AMX, GEN, S3, ERY | Manure | Farm5 |  |
| F-WHQ 1-1 | TET, S3, ERY | Manure | Farm6 |  |
| F-WHQ 1-2 | TET, AMX, CRO, GEN, S3, TIG, ERY | Manure | Farm6 | √ |
| F-WHQ 1-3 | TET, AMX, CRO, GEN, S3, ERY | Manure | Farm6 | √ |
| F-WHQ 2-2 | TET, AMX, S3, ERY | Manure | Farm6 | √ |
| F-WHQ 3-1 | TET, S3, ERY | Manure | Farm6 |  |
| F-WHQ 3-2 | TET, S3, ERY | Manure | Farm6 |  |
| F-WHQ 3-3 | TET, S3, ERY | Manure | Farm6 |  |
| FWJ1-1 | TET, AMX, S3, ERY | Manure | Farm7 |  |
| FWJ1-3 | TET, AMX, S3, ERY | Manure | Farm7 |  |
| FWJ2-3 | TET, AMX, S3, ERY | Manure | Farm7 |  |
| FWJ3-1 | TET, AMX, S3, ERY | Manure | Farm7 |  |
| FWJ3-2 | TET, AMX, S3, FOS, ERY | Manure | Farm7 |  |
| FWJ3-3 | TET, AMX, S3, ERY | Manure | Farm7 |  |
| F-ZZX 1-2 | TET, AMX, S3, ERY | Manure | Farm8 | √ |
| F-ZZX 2-1 | TET, AMX, ERY | Manure | Farm8 | √ |
| F-ZZX 3-1 | TET, ERY | Manure | Farm8 | √ |
| W-FYH 2-2 | TET, AMX, GEN, S3, ERY | Slurry | Farm1 |  |
| W-FYH 2-3 | TET, AMX, GEN, CIP, S3, ERY | Slurry | Farm1 | √ |
| W-FYH 3-1 | TET, S3, ERY | Slurry | Farm1 |  |
| W-HXD 1-1 | TET, S3, ERY | Slurry | Farm2 |  |
| W-HXD 1-2 | TET, AMX, GEN, S3, ERY | Slurry | Farm2 |  |
| W-HXD 1-3 | TET, S3, ERY | Slurry | Farm2 |  |
| W-HXD 2-1 | TET, AMX, S3, TIG, CS, ERY | Slurry | Farm2 | √ |
| W-HXD 2-2 | TET, AMX, S3, CS, FOS, ERY | Slurry | Farm2 | √ |
| W-HXD 3-1 | TET, AMX, S3, ERY | Slurry | Farm2 |  |
| W-HXD 3-3 | TET, AMX, S3, ERY | Slurry | Farm2 | √ |
| W-LJ 1-2 | TET, AMX, S3, ERY | Slurry | Farm4 | √ |
| W-LJ 1-3 | TET, S3, ERY | Slurry | Farm4 | √ |
| W-LJ 2-1 | TET, AMX, CIP, S3, ERY | Slurry | Farm4 | √ |
| W-LJ 2-2 | TET, AMX, S3, ERY | Slurry | Farm4 |  |
| W-LJ 2-3 | TET, AMX, S3, ERY | Slurry | Farm4 |  |
| W-LJ 3-1 | AMX, S3, ERY | Slurry | Farm4 | √ |
| W-LJ 3-2 | TET, AMX, GEN, ERY | Slurry | Farm4 | √ |
| W-LJ 3-3 | TET, GEN, S3, ERY | Slurry | Farm4 | √ |
| W-WHQ 1-1 | TET, AMX, S3, ERY | Slurry | Farm6 |  |
| W-WHQ 1-2 | TET, AMX, CRO, GEN, S3, ERY | Slurry | Farm6 |  |
| W-WHQ 1-3 | TET, AMX, S3, ERY | Slurry | Farm6 |  |
| W-WHQ 2-2 | TET, AMX, S3, ERY | Slurry | Farm6 | √ |
| W-WHQ 2-3 | TET, AMX, S3, ERY | Slurry | Farm6 |  |
| W-WHQ 3-1 | TET, AMX, S3, FOS, ERY | Slurry | Farm6 | √ |
| W-WHQ 3-2 | TET, AMX, CRO, GEN, S3, ERY | Slurry | Farm6 | √ |
| W-WHQ 3-3 | TET, AMX, GEN, S3, FOS, ERY | Slurry | Farm6 | √ |
| W-ZZX 1-1 | S3, ERY | Slurry | Farm8 |  |
| W-ZZX 1-2 | S3, ERY | Slurry | Farm8 | √ |
| W-ZZX 2-2 | AMX, S3, ERY | Slurry | Farm8 | √ |
| W-ZZX 3-1 | TET, S3, ERY | Slurry | Farm8 | √ |
| W-ZZX 3-2 | TET, AMX, S3, ERY | Slurry | Farm8 | √ |
| W-ZZX 3-3 | TET, AMX, S3, ERY | Slurry | Farm8 |  |
| S-FYH 2-1 | TET, AMX, S3, ERY | Fertilized soil | Farm1 | √ |
| S-FYH 3-1 | TET, AMX, S3, ERY | Fertilized soil | Farm1 |  |
| S-FYH 3-2 | TET, S3, ERY | Fertilized soil | Farm1 |  |
| S-HXD 1-1 | TET, AMX, S3, ERY | Fertilized soil | Farm2 |  |
| S-HXD 1-2 | AMX, S3, FOS, ERY | Fertilized soil | Farm2 |  |
| S-HXD 2-1 | TET, AMX, GEN, S3, ERY | Fertilized soil | Farm2 | √ |
| S-HXD 2-2 | TET, AMX, S3, FOS, ERY | Fertilized soil | Farm2 |  |
| S-HXD 2-3 | TET, AMX, CIP, S3, ERY | Fertilized soil | Farm2 | √ |
| S-HXD 3-1 | AMX, ERY | Fertilized soil | Farm2 |  |
| S-HXD 3-2 | TET, AMX, S3, ERY | Fertilized soil | Farm2 |  |
| S-HXD 3-3 | TET, AMX, S3, TIG, FOS, ERY | Fertilized soil | Farm2 | √ |
| S-LJ 1-1 | TET, AMX, S3, ERY | Fertilized soil | Farm4 |  |
| S-LJ 1-2 | TET, AMX, S3, FOS, ERY | Fertilized soil | Farm4 |  |
| S-LJ 1-3 | TET, AMX, S3, ERY | Fertilized soil | Farm4 |  |
| S-LJ 2-1 | AMX, S3, ERY | Fertilized soil | Farm4 |  |
| S-LJ 2-2 | TET, AMX, GEN, S3, TIG, ERY | Fertilized soil | Farm4 | √ |
| S-LJ 2-3 | TET, AMX, GEN, CIP, S3, ERY | Fertilized soil | Farm4 | √ |
| S-LJ 3-1 | TET, AMX, GEN, S3, TIG, FOS, ERY | Fertilized soil | Farm4 | √ |
| S-LKG 1-2 | TET, AMX, GEN, CIP, S3, ERY | Fertilized soil | Farm5 | √ |
| S-LKG 1-3 | TET, AMX, GEN, S3, ERY | Fertilized soil | Farm5 | √ |
| S-LKG 2-1 | AMX, S3, ERY | Fertilized soil | Farm5 |  |
| S-LKG 2-2 | AMX, S3, ERY | Fertilized soil | Farm5 |  |
| S-LKG 2-3 | AMX, S3, ERY | Fertilized soil | Farm5 |  |
| S-LKG 3-1 | AMX, CIP, S3, ERY | Fertilized soil | Farm5 |  |
| S-LKG 3-2 | TET, AMX, S3, ERY | Fertilized soil | Farm5 |  |
| S-LKG 3-3 | TET, AMX, S3, ERY | Fertilized soil | Farm5 |  |
| S-WHQ 1-1 | TET, AMX, GEN, S3, ERY | Fertilized soil | Farm6 |  |
| S-WHQ 1-2 | TET, AMX, GEN, S3, FOS, ERY | Fertilized soil | Farm6 | √ |
| S-WHQ 1-3 | TET, AMX, S3, TIG, ERY | Fertilized soil | Farm6 | √ |
| S-WHQ 2-1 | ERY | Fertilized soil | Farm6 |  |
| S-WHQ 2-2 | ERY | Fertilized soil | Farm6 |  |
| S-WHQ 2-3 | TET, S3, ERY | Fertilized soil | Farm6 |  |
| S-WHQ 3-1 | TET, AMX, S3, FOS, ERY | Fertilized soil | Farm6 |  |
| S-WHQ 3-2 | TET, AMX, S3, ERY | Fertilized soil | Farm6 |  |
| S-WHQ 3-3 | TET, AMX, S3, FOS, ERY | Fertilized soil | Farm6 |  |

Abbreviations: AMX, amoxicillin; CIP, ciprofloxacin; S3, sulfadiazine; ERY, erythromycin; FOS, fosfomycin; TET, tetracycline; CRO, ceftriaxone; GEN, gentamicin; TIG, tigecycline; CS, colistin.

Note: The check mark (√) indicates strains selected for whole-genome sequencing.

**Table S6. Risk assessment of potentially mobile antibiotic resistance genes (ARGs) identified in this study.**

| Potential mobile ARGs | Risk assessment by ARG Ranker (Zhang et al. 2021) |
| --- | --- |
| *AAC(3)-IId* | RankI |
| *AAC(3)-IV* | RankIII |
| *AAC(6')-Ie-APH(2'')-Ia* | RankI |
| *aadA* | RankI |
| *ANT(9)-Ia* | RankIV |
| *APH(3')-Ia* | RankI |
| *APH(3'')-Ib* | RankI |
| *APH(4)-Ia* | RankIV |
| *APH(6)-Id* | RankI |
| *bacA* | RankI |
| *bcrA* | RankIV |
| *bla*_CTX-M-14_ | RankII |
| *emrB* | RankI |
| *emtA* | RankIV |
| *erm(B)* | RankI |
| *erm(C)* | RankI |
| *evgA* | NA |
| *fexA* | RankI |
| *fexB* | RankII |
| *floR* | RankI |
| *gadX* | NA |
| *linG* | RankI |
| *lnu(G)* | RankI |
| *lnuG* | RankI |
| *marA* | NA |
| *mdtG* | NA |
| *mdtK* | RankI |
| *mdtM* | RankI |
| *mefB* | RankI |
| *mel* | RankII |
| *mexM* | RankIV |
| *mph(A)* | RankI |
| *Other streptomycin resistance protein* | RankI |
| *QnrS1* | RankIV |
| *QnrS6* | RankI |
| *QnrS8* | RankI |
| *rosB* | RankIV |
| *sul2* | RankI |
| *sul3* | RankI |
| *bla*_TEM-1_ | RankII |
| *bla*_TEM-126_ | RankIV |
| *bla*_TEM-135_ | RankIV |
| *bla*_TEM-176_ | NA |
| *bla*_TEM-34_ | RankIV |
| *tet(A)* | RankI |
| *tet(B)* | RankI |
| *tet(K)* | RankII |
| *tet(M)* | RankI |
| *tet(X4)* | RankIV |
| *tetM* | RankII |
| *vanH_in_vanP_cl* | NA |
| *vga(C)* | RankIV |

Note: NA indicates the gene was not included in the ARG Risk Ranker database.

**Supplementary** **Text**

**Text S1. Antibiotic Analysis by HPLC-MS/MS**

Enrofloxacin-D5 hydrochloride was used as an internal standard for antibiotic quantification via high-performance liquid chromatography-tandem mass spectrometry (HPLC-MS/MS). Chromatographic separation was achieved using a Zorbax SB-C18 column (50 mm × 2.1 mm, 1.8 µm; Agilent Technologies Inc., CA, USA) maintained at 35°C. The mobile phase consisted of ultrapure water with 0.1% formic acid (Phase A) and acetonitrile (Phase B), delivered at a flow rate of 0.3 mL/min. The gradient elution program was as follows: (a) 0–1 min, A, 90%, B, 10%; (b) 1–2 min, B, 10 → 40%; (c) 2–2.2 min, B, 40 → 70%; (d) 2.2–4 min, B, 70 → 95%; (e) 4–7 min, B, 95%; (f) 7–7.1 min, B, 95 → 10%; (g) 7.1–10 min, B, 10%. Mass spectrometric detection was conducted using positive electrospray ionization (ESI) mode with a source temperature of 350°C. Data acquisition, peak integration, quantification, and calibration curve fitting were performed using Agilent MassHunter software (Version B.07.01). Rigorous quality assurance and quality control (QA/QC) procedures were implemented throughout the analysis, incorporating one solvent blank, one procedural blank, and one standard with a known concentration for background correction and instrument calibration. The method detection limits (MDLs) for all antibiotics ranged from 0.2 to 2 μg/kg in solid-phase samples and from 0.001 to 0.004 μg/L in liquid-phase samples. The method limits of quantitation (LOQs) for each antibiotic ranged from 1 to 4 μg/kg in solid-phase samples and from 0.002 to 0.008 μg/L in liquid-phase samples. Compounds with concentrations below the LOQs were classified as non-detected.

**Text S2. Environmental risk assessment**

2.1 Risk quotient of antibiotics

The risk quotient (RQ) method is commonly used to evaluate the potential ecological risks posed by antibiotics(Menz et al. 2019). For manure and slurry samples, the RQ was calculated by dividing the predicted environmental concentrations (PECs) by the predicted no-effect concentrations (PNECs) (Zhang et al. 2015). For soil samples, the RQ was obtained by dividing the environmental concentrations (ECs) by the PNECs. Depending on the type of PNECs used, the RQ was further categorized to assess potential resistance risks (RQres) and potential toxicity risks (RQtox) using predicted no-effect concentrations for resistance development (PNECres) and toxicity (PNECtox), respectively(Zhang et al. 2015). The RQres for manure, slurry, and soil samples was calculated using Equations (1) and (2), while the RQtox for these samples was determined using Equations (3) and (4) (Wang et al. 2024):

For manure and slurry samples:

$${RQ}_{res-manure/ slurry}=\frac{PEC}{{PNEC}_{res}} (1)$$

$${RQ}_{res-soil}=\frac{EC}{{PNEC}_{res}} (2)$$

For soil samples:

$${RQ}_{tox-manure/ slurry}=\frac{PEC}{{PNEC}_{tox}} (3)$$

$${RQ}_{tox-soil}=\frac{EC}{{PNEC}_{tox}} (4)$$

A nominal RQ classification categorizes risks as follows: RQ < 0.1 predicts low or insignificant risks, 0.1≤RQ≤1 indicates medium risks, and RQ > 1represents high risks to the environment.

Predicted Environmental Concentration (PEC) Calculation:

The predicted environmental concentration (PEC, μg/kg) one year after a single fertilizer application was estimated using the primary exposure model shown in Equation (5):

$$PEC=\frac{C\times M\times(1-w)}{\rho\times D\times10} (5)$$

Where C (μg/kg) is concentration of antibiotics in manure or slurry. *M* is annual manure application rate (dry weight), with a fresh weight of 100 t/ha. 𝜌 is agricultural soil density (1.3 g/cm³). 𝑤 is water content of manure (38.62%). 𝐷 is soil depth (0.2 m).10 is conversion factor.

Predicted No-Effect Concentration (PNEC) Calculation:

PNEC values were derived from the literature or calculated using Equation (6) (Zhang et al. 2015, Bengtsson-Palme and Larsson 2016, Wu et al. 2014):

${PNEC}_{soil}={PNEC}_{water}\times Kd$ (6)

Where *Kd* is the soil–water partition coefficient(Zhang et al. 2015).

A nominal RQ classification of predicts low (or insignificant), medium and high risks in the environment(Wang et al. 2024).

2.2 Risk index of heavy metals

The potential ecological risk index (RI) is a widely used method to evaluate heavy metal pollution in manure, slurry and soils, integrating toxicological and ecological effects. The RI was calculated using Equations (7–9)(Wang et al. 2024).

$C_{f}^{i}=C_{s}^{i}\times C_{n}^{i}$ (7)

$E_{i}=T_{i}\times C_{f}^{i}$ (8)

$RI=\sum E_{I}$ (9)

Where $C_{s}^{i}$ is the concentration of metal i in the samples. $C_{n}^{i}$ denotes the soil background value of metal 𝑖 in Sichuan Province (Table S3). $C_{f}^{i}$is the enrichment coefficient of the heavy metals; Ti is the toxicity response factor, for each metal is defined as follows: As (10), Cd (30), Cr (2), Cu (5), Ni (5), Pb (5), and Zn (1), and Zn (1)(Xiang et al. 2021). Ecological risk index (*E_i_*) quantifies the potential ecological risk of heavy metal 𝑖, categorized into five levels: *Ei <* 40 predicts low risks, 40 ≤ *Ei <* 80 predicts moderate risks, 80 ≤ *Ei <* 160 predicts high risks, 160 ≤ *Ei <* 320 predicts severe risks, *Ei* ≥ 320 predicts very high pollution. RI is classified into five levels: RI *<* 150 predicts none risks, 150 ≤ RI *<* 300 predicts general risks, 600 ≤ RI *<* 1200 predicts high risks, RI *>*1200 predicts very high pollution.

**Text S3. Detail information of *Escherichia coli***

3.1 Isolation and antibiotic resistance of *E. coli*

*E. coli* strains were isolated following the method described by Tan et al. (2024). Briefly, approximately 25 g sample was homogenized with 225 mL of sterile buffered peptone water in an Erlenmeyer flask and incubated at 37°C for 6 h with shaking at 180 rpm. Following pre-enrichment, 1 mL of the culture was transferred into 9 mL of Enterobacteriaceae Enrichment (EE) broth and incubated at 37°C for 24 ± 2 h. A loopful of EE broth was streaked onto eosin methylene blue (EMB) agar and incubated at 37°C for 24 ± 2 h. Presumptive E. coli colonies, characterized by a green metallic sheen under reflected light and a dark center under transmitted light, were subcultured on tryptic soy agar (TSA) and incubated overnight at 37°C. Three colonies per sample were preserved in tryptic soy broth (TSB) with 20% glycerol at –80°C. *E. coli* identification was confirmed via 16S rRNA gene sequencing. Subsequently, the identified *E. coli* isolates were subjected to antimicrobial susceptibility testing to evaluate their drug resistance profiles.

The minimum inhibitory concentrations (MICs) of *E. coli* were determined using the agar dilution method in accordance with Clinical and Laboratory Standards Institute (CLSI) guidelines. Twelve antibiotics were tested, including tetracycline, amoxicillin, ceftriaxone, aztreonam, gentamicin, ciprofloxacin, sulfadiazine, tigecycline, colistin, fosfomycin, erythromycin, and imipenem. The resistance phenotypes of *E. coli* are provided in Table S5.

3.2 Selection of resistant *E. coli*

Resistant *E. coli* strains were isolated from manure, slurry, and fertilized soil, and a total of 39 resistant *E. coli* strains were selected based on their sources and resistance phenotypes (Table S5). For isolations from the same sample, strains exhibiting distinct resistance phenotypes were prioritized. When multiple isolates from one sample displayed varied resistance patterns, those demonstrating the broadest spectrum of resistance were chosen, with a maximum of three isolates selected per sample.

**Text S4. Health risk assessment**

The health risks associated with antibiotic resistance genes (ARGs) in manure, slurry, and soil were comprehensively assessed based on ARG abundance, mobility (MO), host pathogenicity (HP), human accessibility (HA), and clinical availability (CA), with modifications based on previous studies(Zhang et al. 2022). The values for HA and CA were obtained from the literature(Zhang et al. 2022). Mobility (MO) represents the ability of ARGs to transfer between hosts via horizontal gene transfer (HGT). The MO of ARGs was determined by the number of associated mobile genetic elements (MGEs) identified in genomes, which could potentially mediate their transfer between genomes or to plasmids. Host pathogenicity (HP) specifically reflects the likelihood of ARGs transferring from non-pathogenic to pathogenic hosts. This process may lead to the evolution of antibiotic-resistant pathogens, compromising the efficacy of clinical infection control. HP for each ARG was calculated using the following formula:

*HP*$={Number}_{pathogenic}/{Number}_{all}$

where Number_pathogenic_ is the number of pathogenic ARG hosts and Number_all_ is the total number of hosts containing ARGs.

The risk index (RI) of ARGs to human health was then calculated as:

$$RI=MO\times HP\times HA\times CA$$

To calculate the overall health risk for each sample, the following formula was applied:

$${RI}_{sample}=\sum_{i=1}^{n} {Abundance}_{i}\times{RI}_{i}$$

where Abundance_i_ is the relative abundance of ARG i in the sample, and *RI_i_* is the RI of ARG i.

**Reference**

Zhao, Y., Chen, Y.P., Zheng, Y., Ma, Q. and Jiang, Y. (2020) Quantifying the heavy metal risks from anthropogenic contributions in Sichuan panda (*Ailuropoda melanoleuca melanoleuca*) habitat. Sci. Total. Environ 745, 140941.

Zhang, A.-N., Gaston, J.M., Dai, C.L., Zhao, S., Poyet, M., Groussin, M., Yin, X., Li, L.-G., van Loosdrecht, M.C.M., Topp, E., Gillings, M.R., Hanage, W.P., Tiedje, J.M., Moniz, K., Alm, E.J. and Zhang, T. (2021) An omics-based framework for assessing the health risk of antimicrobial resistance genes. Nature Communications 12(1), 4765.

Menz, J., Olsson, O. and Kümmerer, K. (2019) Antibiotic residues in livestock manure: Does the EU risk assessment sufficiently protect against microbial toxicity and selection of resistant bacteria in the environment? Journal of Hazardous Materials 379, 120807.

Zhang, Q.-Q., Ying, G.-G., Pan, C.-G., Liu, Y.-S. and Zhao, J.-L. (2015) Comprehensive Evaluation of Antibiotics Emission and Fate in the River Basins of China: Source Analysis, Multimedia Modeling, and Linkage to Bacterial Resistance. Environmental Science & Technology 49(11), 6772-6782.

Wang, X., Zhang, X., Li, N., Yang, Z., Li, B., Zhang, X. and Li, H. (2024) Prioritized regional management for antibiotics and heavy metals in animal manure across China. Journal of Hazardous Materials 461, 132706.

Bengtsson-Palme, J. and Larsson, D.G.J. (2016) Concentrations of antibiotics predicted to select for resistant bacteria: Proposed limits for environmental regulation. Environment International 86, 140-149.

Wu, X.-L., Xiang, L., Yan, Q.-Y., Jiang, Y.-N., Li, Y.-W., Huang, X.-P., Li, H., Cai, Q.-Y. and Mo, C.-H. (2014) Distribution and risk assessment of quinolone antibiotics in the soils from organic vegetable farms of a subtropical city, Southern China. Science of the Total Environment 487, 399-406.

Xiang, M., Li, Y., Yang, J., Lei, K., Li, Y., Li, F., Zheng, D., Fang, X. and Cao, Y. (2021) Heavy metal contamination risk assessment and correlation analysis of heavy metal contents in soil and crops. Environmental Pollution 278, 116911.

Blanco-Míguez, A., Beghini, F., Cumbo, F., McIver, L.J., Thompson, K.N., Zolfo, M., Manghi, P., Dubois, L., Huang, K.D., Thomas, A.M., Nickols, W.A., Piccinno, G., Piperni, E., Punčochář, M., Valles-Colomer, M., Tett, A., Giordano, F., Davies, R., Wolf, J., Berry, S.E., Spector, T.D., Franzosa, E.A., Pasolli, E., Asnicar, F., Huttenhower, C. and Segata, N. (2023) Extending and improving metagenomic taxonomic profiling with uncharacterized species using MetaPhlAn 4. Nature Biotechnology 41(11), 1633-1644.

Urban, M., Cuzick, A., Rutherford, K., Irvine, A. and Pedro, H. (2017) PHI-base: a new interface and further additions for the multi-species pathogen-host interactions database. 45(D1), D604-d610.

Pärnänen, K., Karkman, A. and Hultman, J. (2018) Maternal gut and breast milk microbiota affect infant gut antibiotic resistome and mobile genetic elements. 9(1), 3891.

Li, X., Rensing, C., Vestergaard, G., Arumugam, M., Nesme, J., Gupta, S., Brejnrod, A.D. and Sørensen, S.J. (2022) Metagenomic evidence for co-occurrence of antibiotic, biocide and metal resistance genes in pigs. Environment International 158, 106899.

Zhang, Z., Zhang, Q., Wang, T., Xu, N., Lu, T., Hong, W., Penuelas, J., Gillings, M., Wang, M., Gao, W. and Qian, H. (2022) Assessment of global health risk of antibiotic resistance genes. Nature Communications 13(1), 1553.
